# Crowdsourcing interventions to promote uptake of COVID-19 booster vaccines

**DOI:** 10.1016/j.eclinm.2022.101632

**Published:** 2022-09-05

**Authors:** Robert Böhm, Cornelia Betsch, Yana Litovsky, Philipp Sprengholz, Noel T. Brewer, Gretchen Chapman, Julie Leask, George Loewenstein, Martha Scherzer, Cass R. Sunstein, Michael Kirchler

**Affiliations:** a Faculty of Psychology, University of Vienna, Austria; b Department of Psychology, University of Copenhagen; 1354 Copenhagen K, Denmark; c Copenhagen Center for Social Data Science (SODAS), University of Copenhagen, Denmark; d Media and Communication Science, University of Erfurt, Germany; e Center for Empirical Research in Economics and Behavioral Sciences (CEREB), University of Erfurt, Germany; f Bernhard Nocht Institute for Tropical Medicine (BNITM), Hamburg, Germany; g Department of Banking and Finance, University of Innsbruck, Austria; h Department of Health Behavior, Gillings School of Global Public Health and Lineberger Comprehensive Cancer Center, University of North Carolina at Chapel Hill, USA; i Department of Social and Decision Sciences, Carnegie Mellon University, USA; j Faculty of Medicine and Health, The University of Sydney, Australia; k Consultant, Behavioral and Cultural Insights Unit, WHO Regional Office for Europe, Copenhagen, Denmark; l Harvard Law School, Harvard University, USA

**Keywords:** Booster vaccination, COVID-19, Behavioral interventions, Crowdsourcing

## Abstract

**Background:**

COVID-19 booster vaccine uptake rates are behind the rate of primary vaccination in many countries. Governments and non-governmental institutions rely on a range of interventions aiming to increase booster uptake. Yet, little is known how experts and the general public evaluate these interventions.

**Methods:**

We applied a novel crowdsourcing approach to provide rapid insights on the most promising interventions to promote uptake of COVID-19 booster vaccines. In the first phase (December 2021), international experts (*n* = 78 from 17 countries) proposed 46 unique interventions. To reduce noise and potential bias, in the second phase (January 2022), experts (*n* = 307 from 34 countries) and representative general population samples from the UK (*n* = 299) and the US (*n* = 300) rated the proposed interventions on several evaluation criteria, including effectiveness and acceptability, on a 5-point Likert-type scale.

**Findings:**

Sanctions were evaluated as potentially most effective but least accepted. Evaluations by expert and general population samples were considerably aligned. Interventions that received the most positive evaluations regarding both effectiveness and acceptability across evaluation groups were: a day off work after getting vaccinated, financial incentives, tax benefits, promotional campaigns, and mobile vaccination teams.

**Interpretation:**

The results provide useful insights to help governmental and non-governmental institutions in their decisions about which interventions to implement. Additionally, the applied crowdsourcing method may be used in future studies to retrieve rapid insights on the comparative evaluation of (health) policies.

**Funding:**

This study received funding from the Austrian Science Fund (SFB F63) and the University of Vienna.

**Research in context**

**Evidence before this study**

We searched Medline (through PubMed) and Web of Science until December 2021 (before the study was conducted), using different combinations of the following keywords: booster, vaccin[truncation symbol], covid[truncation symbol], and intervention. We found some studies investigating the willingness to receive a booster vaccination, but no studies investigating potential interventions to increase COVID-19 booster vaccine uptake. We extended the search until June 2022 (when revising the manuscript) and additionally searched for unpublished manuscripts on Google Scholar. We found some studies (described in the main text) investigating interventions on COVID-19 booster uptake intention, yet, there was no study comparing many interventions simultaneously, or including evaluations by both experts and participants from the general population.

**Added value of this study**

This study uses a crowdsourcing approach (1) to solicit existing interventions worldwide and propose new ones to increase COVID-19 booster vaccine uptake, and (2) to assess the perceived effectiveness and acceptability (among other evaluation criteria) of these interventions. Experts from various disciplines (but mainly researchers and practitioners with a background in social/economic science or public health) were asked to propose interventions of any kind. The interventions were then evaluated on various criteria by both experts and people from the general population in the UK and US. The study thus contributes to the existing literature by specifically targeting known and novel interventions to the context of COVID-19 booster vaccination. The study further advances the existing knowledge by providing comparisons—and identifying the most promising interventions—across several evaluation criteria and evaluation groups (i.e., experts and the general population).

**Implications of all the available evidence**

This study adds evidence that can be used by governmental and non-governmental institutions to identify potentially effective and acceptable interventions (or based on other criteria), aimed at increasing COVID-19 booster vaccine uptake. Given that evaluations may differ across time, evaluation group, and target population, the interventions should be adapted, contextualized, and tested for actual effectiveness in order to be applied in different settings. The study design provides a blueprint for generating and evaluating interventions in the context of COVID-19 vaccination and beyond.

Alt-text: Unlabelled box

## Introduction

Achieving high coverage for COVID-19 vaccination globally is the most important action to reduce hospitalizations and death. Immunity begins to wane only a few months or even weeks after primary vaccination^1^ and booster vaccination (i.e., additional vaccine doses after primary vaccination) is becoming routine to increase the effectiveness of the vaccines against infection and particularly severe disease.^2^^,^^3^ Amidst the global spread of the Omicron variant, its lineages and sub-lineages, many countries have been rolling out COVID-19 booster vaccines to the general adult population since the middle of 2021, and some have announced plans or do already offer a second booster dose (e.g., Australia, Israel, Chile, and the UK). Despite being recommended in many countries, as of July 11, 2022, only 74% of the fully vaccinated (without booster) have received a booster vaccination in the European Union, and rates are even lower in Australia (64%), South America (64%), North America (58%), Asia (41%) and Africa (11%).^4^ While this discrepancy is in part due to insufficient supply of and access to COVID-19 vaccines, some previously vaccinated people are unwilling or hesitant to get the booster vaccination, even in countries with initially high COVID-19 vaccine uptake.^5^, ^6^, ^7^, ^8^ To increase uptake of booster vaccines, most countries inform people about the benefits of boosters, and some countries also employ interventions like sending personal reminders (e.g., Denmark, UK), offering incentives (e.g., Lithuania, many US states), imposing various restrictions on those who have not been boosted (e.g., France, Germany), or even imposing mandates with financial sanctions (e.g., Greece, Malaysia). In addition, non-governmental institutions and companies have adopted similarly heterogenous approaches to increase COVID-19 booster vaccine uptake of their workforce, including financial incentives^9^ and mandates.^10^

This heterogeneity in implemented interventions may in part be rooted in different epidemiological situations, healthcare systems, and vaccination programs. It may, however, also be due to the lack of evidence about which kind of interventions effectively increase COVID-19 booster vaccine uptake and reliance on a small number of advisors (if any), creating a risk of undue reliance on individual opinions when imposing nationwide or company-wide interventions. A substantial evidence base is available to guide decisions about increasing vaccine uptake in general,^11^ however, relevant data on the effectiveness of interventions in the novel situation of promoting COVID-19 booster vaccination will be delayed and cannot be used when it is needed—now. Although there have been some first empirical studies investigating the potential effectiveness of selected interventions on COVID-19 booster uptake,^12^ it is yet too early to know the relative effectiveness of the interventions that are presently being implemented in different settings as suggested by different groups of experts.

The aim of the present research is to provide insights into which interventions are perceived to be most effective and acceptable (among other criteria) to increase uptake of COVID-19 boosters. To circumvent the challenges associated with only a few advisors, we rely on a large-scale crowdsourcing approach—the process of aggregating individual opinions to solve a problem—to generate insights about which interventions are most promising. However, the successful implementation of interventions depends on various factors, and even expert opinions may not necessarily be accurate when making single point subjective estimates about an intervention's overall effectiveness.^13^^,^^14^ For example, interventions also need to be accepted by the general population or the employees,^15^, ^16^, ^17^ and evidence about the acceptability of different interventions to promote COVID-19 booster uptake is also lacking. Instead of asking whether experts and people from the general population may have different expertise in their evaluation of relevant criteria, in this paper, we assess how much both groups agree on evaluating potential interventions and whether there are some interventions that both groups regard as effective and acceptable. We therefore aim to reduce both noise and potential bias by relying on independent subjective evaluations from experts as well as the general population, without enforcing agreement in the evaluation of interventions within or between evaluation groups.

## Method

**Figure 1:**
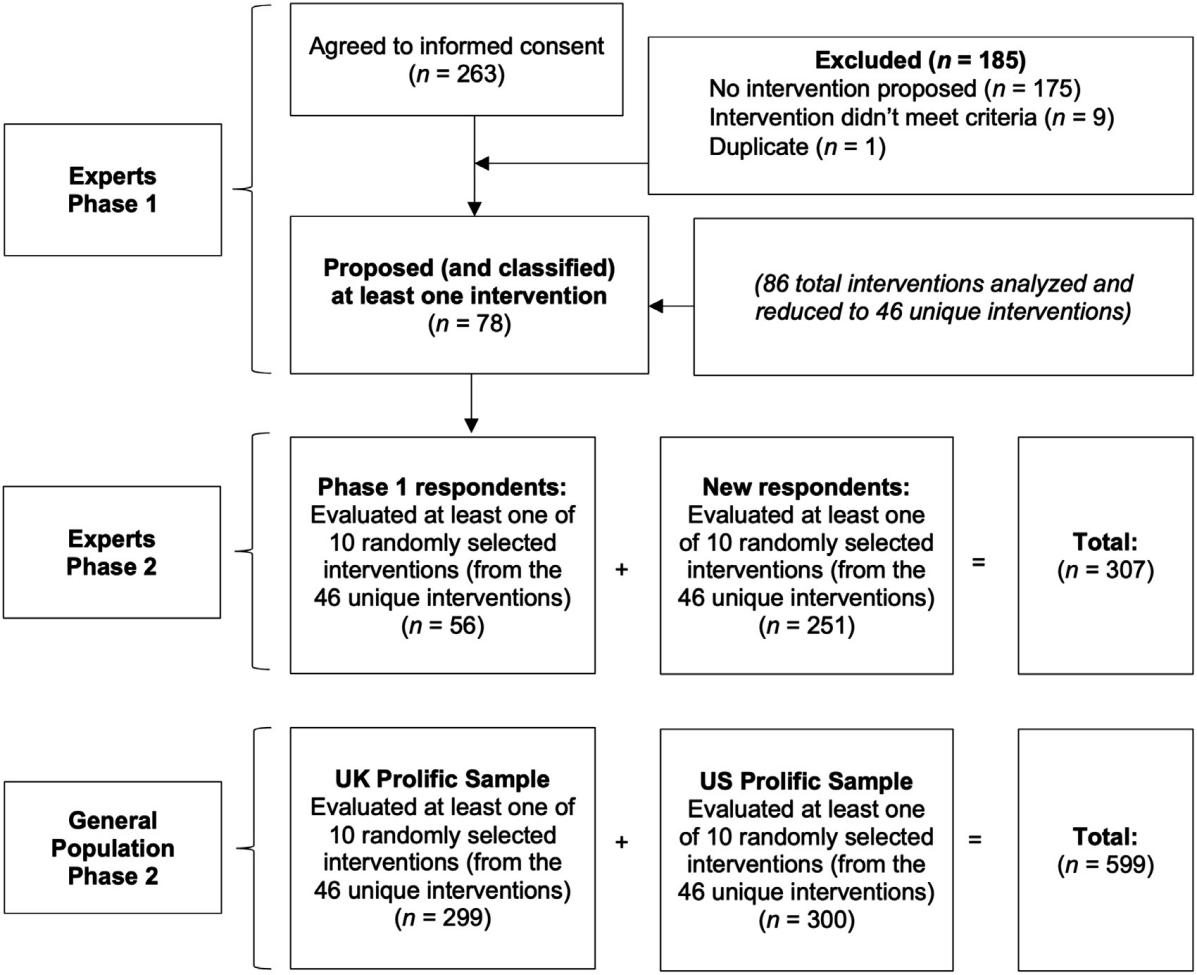
Flow chart of the different phases of the study, including sample types and sizes as well as tasks.

This exploratory study is composed of two phases, each with interrelated online surveys among different samples. Figure 1 provides an overview of the overall study flow. Below we describe each of the phases separately in a chronological order, including its samples, procedures, and measures.

### Ethics and open practices

This study received ethical clearance from the Institutional Review Board of the Department of Occupational, Economic, and Social Psychology at the Faculty of Psychology, University of Vienna, Austria (project number: 2021/W/001). All participants provided informed consent. We did not expect any harm for study participants. The study was pre-registered via the Open Science Framework (https://osf.io/94ugm; original pre-registration: 2021-12-08, amendment: 2022-01-11).

### Phase-one survey

#### Sample

In December 2021—when booster vaccines were announced or made available in many countries—experts from various disciplines were invited to propose interventions that could potentially increase uptake rates of COVID-19 booster vaccines. The convenience sample of experts was recruited via email lists that were considered appropriate to recruit practitioners and researchers working on vaccination behavior, or social and behavioral scientists more generally, i.e., Behavioral Insights Community of Practice by the World Health Organization/Regional Office for Europe, Collaboration on Social Science and Immunization (COSSI), Economic Science Association (ESA), German Association of Psychology (DGPs), and Society of Judgment and Decision Making (JDM). Invitations were sent in calendar week 49, 2021, and participants were asked to complete the survey within one week. All people who were signed-up for either of the email lists were eligible to participate; there were no exclusion criteria. All interventions proposals that were explained in sufficient detailed according to the task description (see below) were further processed. Overall, *n* = 78 scientists and practitioners from the social and behavioral sciences, medical sciences, and population health sciences from 17 countries in Asia, Australia, Europe, North and South America (*n* = 3 participants from middle-income countries, such as Brazil, China, and Malaysia; *n* = 72 participants from high-income countries, such as Germany, the UK and US; *n* = 3 with not response) participated in the survey. Experts had a mean of 17 years of self-reported professional expertise in their fields (for further sample characteristics, see Table 1).

**Table 1:** Characteristics of samples.

Variable	Experts (phase-one survey, *n* = 78)	Experts (phase-two survey, *n* = 307)	General population sample UK (phase-two survey, *n* = 299)	General population sample US (phase-two survey, *n* = 300)
Gender: %				
Female	48·72%	38·11%	50·83%	51·00%
Male	47·44%	42·02%	49·17%	49·00%
Non-binary	0·00%	0·65%	0·00%	0·00%
No response	3·84%	19·22%	0·00%	0·00%
Age: mean (SD)	42·92 (12·38)	3·33 (11·2)	44·9 (15·53)	45·21 (16·17)
Disciplines: %				
Medicine or Health Care	6·41%	2·61%	NA	NA
Economics	29·49%	31·60%	NA	NA
Public Health	3·85%	3·58%	NA	NA
Psychology	46·15%	31·92%	NA	NA
Other	10·26%	12·05%	NA	NA
Experience in years: mean (SD)	17·12 (11·98)	13·66 (10·43)	NA	NA
Education: %				
Less than high school	NA	NA	0·33%	1·33%
High school or equivalent	NA	NA	26·76%	11·00%
Some college	NA	NA	37·79%	45·00%
Post-graduate education	NA	NA	35·12%	42·67%
Political attitude, mean (SD)	NA	NA	4·57 (1·49)	4·89 (1·73)
Libertarian morality, mean (SD)	NA	NA	3·20 (0·51)	3·08 (0·53)

Notes. **Gender**: female, male, non-binary, prefer not to say. **Age**: numeric response in years (18-99). **Discipline**: Listed options. **Experience in years:** Number of years working in the field (after first university diploma/degree). **Education:** Listed options. **Political Attitude:** Likert scale response: (1) Very conservative, (2) Moderately conservative, (3) Slightly conservative, (4) Neither liberal nor conservative, (5) Slightly liberal, (5) Moderately liberal, (6) Very liberal. **Libertarian moral values:** Likert Scale from (1) Strongly disagree to (5) Strongly agree used to evaluate 3 statements: (1) Society works best when it lets individuals take responsibility for their own lives without telling them what to do. (2) The government interferes far too much in our everyday lives. (3) The government should do more to advance the common good, even if that means limiting the freedom and choices of individuals. **NA:** variable was not assessed for this sample. Percentages of disciplinary affiliation do not add up to 100% because of missing values.

#### Procedure and measures

The survey was programmed with Qualtrics. Experts proposed interventions that could be implemented by governments, health organizations, companies, or other agencies, and were asked to describe them in sufficient detail such that they could inform actual interventions in practice. In detail, participants were asked to propose interventions using the following instructions: “*Please propose one intervention that can be implemented by governments, agencies, or health organizations and that is, in your view, most effective and feasible to increase uptake of COVID-19 booster vaccines in the country where you work. In this case, we define ‘intervention’ as a planned and focused activity aiming at increasing booster vaccine uptake, specifically: The intervention aims to increase uptake of boosters for adults. Therefore, the intervention should focus on adults (age 18+) for whom a booster is recommended in the country where you work. Please describe the intervention with key implementation information: What would the intervention look like in reality? Imagine you or your organization would be the implementers of this—provide the information necessary to make the intervention work. Examples of potential questions you might address include: What procedures does the intervention change compared to the status quo? How, when, and where is the intervention implemented? Who implements the intervention? What are further details that a person or organization implementing the intervention would need to know? Later in the survey, you will have the opportunity to classify and rate the likely effectiveness of the proposed intervention. Please describe only one intervention at a time. If you wish, you will be able to add more interventions later.*”

After participants had described their intervention proposals, they were asked to classify each intervention according to various criteria to better understand what intervention processes they aimed to address. Classification criteria were similar to those from the Behavior Change Wheel^18^ (see Table 2, top panel, and Survey Materials on OSF). Next, participants were asked to evaluate the interventions they proposed on criteria adapted from the APEASE criteria by the Behavior Change Wheel (Table 2, bottom panel). In contrast to the original criteria, we asked to evaluate acceptability of the intervention to both stakeholders and eligible adults. Further, we added two criteria of relevance to the present context: universality across different countries and effect on unvaccinated people. In case practicability was rated <5 and non-pharmaceutical side effects were rated >1, participants were asked to briefly describe potential barriers and unintended non-pharmaceutical effect, respectively (open text response). Finally, participants were also asked to provide some demographic information: gender, age, profession, discipline, country in which they work, years of experience after university degree. They were also able to leave comments, their name (to be acknowledged), and their email address to be contacted for the phase-two survey.

**Table 2:** Classification and evaluation criteria assessed in the surveys.

Classification criteria		
Criterion	Definition	
**Education**	Increasing understanding of the disease, the vaccine or how to get vaccinated	
Persuasion	Using communication to change what people think or feel	
Modeling	Providing an example for people to aspire to or imitate	
Psychological enablement	Increasing the likelihood of people turning positive intentions intro behavior (e.g., nudging)	
Environmental restructuring	Changing the physical context where vaccinations take place	
Incentivization	Providing positive reward for vaccination	
Restriction	Restrict the opportunity to engage in other desirable behaviors if unvaccinated	
Sanction	Creating expectation of punishment or financial cost if unvaccinated	
Evaluation criteria		
Criterion	Definition	Scale (1-5)
Affordability*	How costly (financially) do you think the intervention is for the implementing governments, agencies, or health organizations compared to other potential interventions?	‘Very cheap’ to ‘Very costly’
Practicability*	Can the intervention be delivered as intended for eligible adults?	‘Definitely not’ to ‘Definitely’
Effectiveness*^,^‡	How much will the intervention increase uptake of COVID-19 booster vaccination in a real-world context?	‘Not at all’ to ‘Very much’
Effectiveness for self‡	How much will the intervention increase your likelihood of getting the COVID-19 booster vaccination?	‘Not at all’ to ‘Very much’
Acceptability to stakeholders*	How likely are the people who would implement the intervention (e.g., political decision makers, community leaders, health workers) to accept it (e.g., not protesting against it)?	‘Very unlikely’ to ‘Very likely’
Acceptability to eligible adults*^,^‡	How likely are adults eligible for COVID-19 vaccine boosters to accept this intervention (i.e., not protesting against it)?	‘Very unlikely’ to ‘Very likely’
Non-pharmaceutical side effects*	Will there be any potential unintended outcomes of the intervention?	‘Definitely not’ to ‘Definitely’
Inequities*	How will the intervention affect social and health inequalities in adult COVID-19 vaccine booster uptake?	‘Definitely decrease inequalities’ to ‘Definitely increase inequalities’
Universality*	Please indicate whether you believe the proposed intervention is appropriate universally across different countries. With appropriateness we mean both feasibility and effectiveness.	‘Specific to a certain country or region of the world’ to ‘Universally appropriate’
Effect on unvaccinated*	Although COVID-19 booster vaccines are for people already fully vaccinated, do you anticipate any effect of the proposed intervention on unvaccinated people?	‘Definitely decrease their vaccine uptake’ to ‘Definitely increase their vaccine uptake’
Coerciveness‡	How coercive is this intervention?	‘Not at all’ to ‘Very much’
Reactance‡	To what extent do you perceive the intervention as a restriction of your freedom? Would you be frustrated about the intervention? How much would the intervention annoy you? To what extent would you be offended/disturbed by the intervention?	‘Not at all’ to ‘Very much’
Activism‡	How likely would you be to sign a petition against the intervention? How likely would you be to take part in a demonstration against the intervention? How likely would you be to join a lawsuit against the intervention? How likely would you be to encourage others to join in efforts against the intervention?	Very unlikely’ to ‘Very likely’

Note.

⁎ Evaluated by expert sample.

‡ Evaluated by general population samples. For all items, the midpoint (3) was pre-selected on the slider.

#### Selection and classification of intervention proposals

Three independent raters from the author team read the proposed interventions and evaluated which proposals are sufficiently similar to be merged. Rater disagreement was solved by discussion. From the overall 86 intervention proposals we received, we identified 46 unique interventions. Whereas most interventions targeted the general population, some interventions only targeted specific subpopulations, such as vulnerable persons. Descriptions were adjusted to be comparable in length and language style; we also provided a short title for each intervention. Next, two independent raters from the author team classified each unique intervention according to the evaluation criteria adapted from the Behavior Change Wheel.^18^ In contrast to the original classification criteria, we removed the category ‘Training’ because we saw little fit to the present context. All other criteria were adapted to the respective context, that is, interventions to promote uptake of COVID-19 booster vaccines (see Table 2, top panel). Each intervention was assigned to at least one category. Rater disagreement was solved by discussion. Table S1 in the Supplementary Material provides an overview of all unique interventions and their classification.

### Phase-two survey

#### Sample

In the second phase, we invited the same experts who participated in the phase-one survey and additional experts via the same mailing lists as used for disseminating the phase-one survey. Invitations were sent in calendar week 2, 2022, and participants were asked to complete the survey within one week. Overall, the responding experts (*n* = 307) came from 34 countries in Africa, Asia, Australia, Europe, North and South America (*n* = 16 participants from middle-income countries, such as India, Peru, and South Africa; *n* = 233 participants from high-income countries, such as Canada, France, and Israel; *n* = 58 with no response). Experts had a mean of 14 years of self-reported professional expertise in their fields (for further sample characteristics, see Table 1). Among all participants, we distributed 20 $100 prizes to be given to randomly chosen participants who completed the survey (either for personal payment or donation to a charity of their choice).

Additionally, we recruited two other samples from the general population, i.e., people for whom booster vaccination had been recommended. Participants were recruited via the online panel recruitment company Prolific (https://www.prolific.co/). We used Prolific's built-in feature to invite samples from the UK and US general adult population, quota-representative for age, gender, and ethnicity. The UK and US were chosen because (1) both countries had insufficient COVID-19 booster uptake at the time of the study and (2) it was considered feasible to recruit fairly heterogenous and quota-representative (online) samples within a rather short study period with English survey materials. We recruited *n* = 299 participants from the UK (there was one respondent less than requested due to some technical problems) and *n* = 300 participants from the US (for sample characteristics, see Table 1). Each participant received remuneration of £1.50 for completion of the study.

#### Procedure and measures

The survey was programmed with Qualtrics. Each respondent evaluated a random subset of 10 interventions, leading to, on average, 57 expert ratings and 130 ratings by people from the general population per intervention. Experts were asked to evaluate the intervention proposals on nine criteria adapted and extended from the Behavior Change Wheel's APEASE criteria (see phase-one survey).^18^ Respondents from the general population were asked to evaluate the intervention proposals on a subset of the criteria that were considered relevant and easy for the general public to answer. Additionally, they were asked to answer additional questions that were of particular relevance to the target population, such as the perceived effectiveness in increasing their personal likelihood of having a booster vaccination, perceived coerciveness, psychological reactance (four items adapted from the Salzburger State Reactance Scale;^19^ Cronbach's α = ·95), as well as intentions to actively oppose the intervention if it would be implemented (four items adapted from Sprengholz et al.;^20^ Cronbach's α = ·93). All measures, including their respective items and response scale, are summarized in Table 2, bottom panel. Participants from the general population also completed measures assessing their libertarian moral values (three items adapted from Iyer et al.,^21^ e.g., ‘The government interferes far too much in our everyday lives.’; 1 = ‘strongly disagree’ to 7 = ‘strongly agree’; Cronbach's α = ·75) and political attitude (one item, ‘What is your general political attitude?’; 1 = ‘very liberal’ to 7 = ‘very conversative’). Finally, participants were asked to provide some demographic information: gender, age, education (only general population), profession (only experts), discipline (only experts), country in which they work (only experts), years of experience after university degree (only experts).

### Statistical analyses

In a first step, we analyzed subjective evaluations by intervention classes. We conducted mixed effects regressions—separately for each evaluation criterion as well as for experts and participants from the general population—with all intervention classes as predictors. Given that each participant evaluated several intervention proposals, we consider the participant as a random effect to control for the interrelated error variance. We report the 95% confidence intervals of the unstandardized regression coefficients; an effect with an interval not including zero is considered significant. The full regression “base models” are reported in the Supplementary Material, where we also report “extended models” with several characteristics of the evaluators as covariates (the results remain qualitatively identical). To analyze potential relations between different perceptions of intervention proposals, we further report selected 95% confidence intervals from Pearson correlation coefficients (all zero-order correlations are reported in the Supplementary Material).

In a second step, we analyzed the subjective evaluations of single interventions. Specifically, to quantify the level of (mis)alignment in evaluations by experts and respondents from the general population, we calculated the correlation between the mean ratings by evaluation group across all 46 interventions, separately for each of the two criteria that were evaluated by both groups: effectiveness and acceptability.

### Role of funding source

The funding sources had no role in designing the study; analyzing or interpreting the data. The raw data was not accessible to the funders; the raw data was accessed and analyzed by R.B., C.B., Y.L., P.S., and M.K. All authors have overseen and agreed to the interpretation of results. All authors have approved the submission.

## Results

### Evaluation of intervention classes

**Figure 2:**
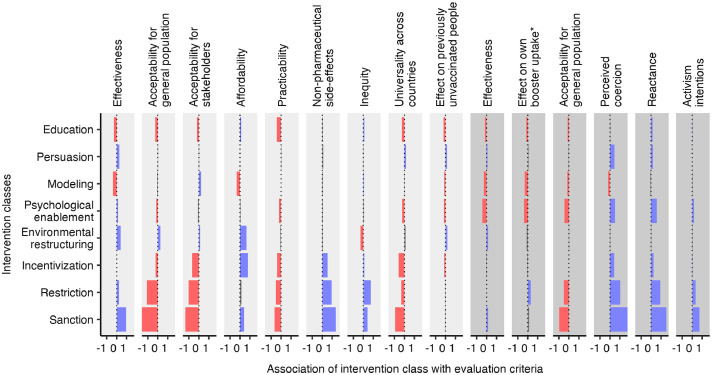
Evaluation of intervention classes. Colored bars represent unstandardized regression coefficients with a negative (red) or positive (blue) sign being different from zero (*p* < ·05) by experts (*n* = 307; light gray) and respondents from the general population (*n* = 599; dark gray), respectively (see Tables S2-S16 in the Supplementary Material for details on these regression analyses and extended analyses controlling for participants’ age and gender as well as experts’ profession, discipline, and participation history (i.e., if they provided data in both phases or just phase 2). *Based on a subsample of participants who have not yet received a booster vaccine at the time of the study (*n* = 144).

The most prevalent intervention classes among all proposed interventions in the first phase were education (50% of all interventions), persuasion (33%), modeling (30%), and psychological enablement (30%) (for a complete list, see Supplementary Material). According to experts’ evaluation, no intervention class was best on all evaluation criteria (Figure 2). Perceived effectiveness was most positively predicted for interventions relying on sanctions (i.e., creating expectation of punishment or financial cost if unvaccinated; unstandardized regression coefficient: 95% CI [0·79, 1·14]). Regarding acceptability to both stakeholders (e.g., political decision makers, community leaders, health workers; 95% CI [−1·55, −1·20]) and to the general population (95% CI [−1·77, −1·43]), however, sanctions were evaluated most negatively, closely followed by restrictions (stakeholders: 95% CI [−1·24, −0·81]; general population: 95% CI [−1·30, −0·88]). This is also captured by experts’ expectations that interventions relying on sanctions, restrictions (i.e., restricting the opportunity to engage in other desirable behaviors if unvaccinated), or incentives (i.e., providing positive reward for vaccination) might cause non-pharmaceutical (i.e., social and psychological) side effects (sanctions: 95% CI [1·20, 1·54]; restrictions: 95% CI [0·74, 1·16]; incentives: 95% CI [0·43, 0·66]) and increase health inequalities (sanctions: 95% CI [0·27, 0·56]; restrictions: 95% CI [0·57, 0·92]; incentives: 95% CI [0·02, 0·22]). Only interventions relying on environmental restructuring (i.e., changing the physical context where vaccinations take place) were expected to increase the acceptability to the general population (95% CI [0·14, 0·40]) and decrease health inequalities (95% CI [−0·39, −0·18]), but were considered relatively ineffective by the experts (95% CI [0·27, 0·54]).

These findings are largely mirrored by the evaluations provided by the respondents from the general population. Sanctioning interventions were evaluated, among all intervention classes, as the most likely to increase booster uptake in the general population (95% CI [0·05, 0·27]). However, among vaccinated respondents who have not yet received a booster (*n* = 144), only restrictions were expected to increase their own likelihood of getting a booster vaccination. Yet, sanctions (95% CI [−1·08, −0·86]) and restrictions (95% CI [−0·65, −0·38]) were deemed as least acceptable.

### Evaluation of single interventions

Examining the correlations between the evaluation criteria across all interventions (for a complete correlation matrix, see Supplementary Material), it is noteworthy that experts perceived only a weak positive relationship between an intervention's acceptability for the general population and its effectiveness (Pearson correlation: 95% CI [·08, ·16]). In contrast, the positive link between acceptability and effectiveness was perceived to be much stronger by respondents from the general population (95% CI [·35, ·44]). The latter effect might be explained by the fact that lower expected acceptability was also associated with respondents from the general public anticipating larger psychological reactance (95% CI [−·35, −·30]) and more activism intentions against the intervention (95% CI [−·23, −·18]).

Regarding the consensus in evaluations, evaluations regarding interventions’ expected overall effectiveness from experts and people from the general population correlated substantially (95% CI [·29, ·71]). Similarly, experts and the general population also had high agreement regarding the interventions’ acceptability (95% CI [·60, ·85]). However, experts’ evaluations of expected overall effectiveness and the general population's own likelihood of getting the booster vaccine was not significantly correlated (95% CI [−·09, ·47]).

**Figure 3:**
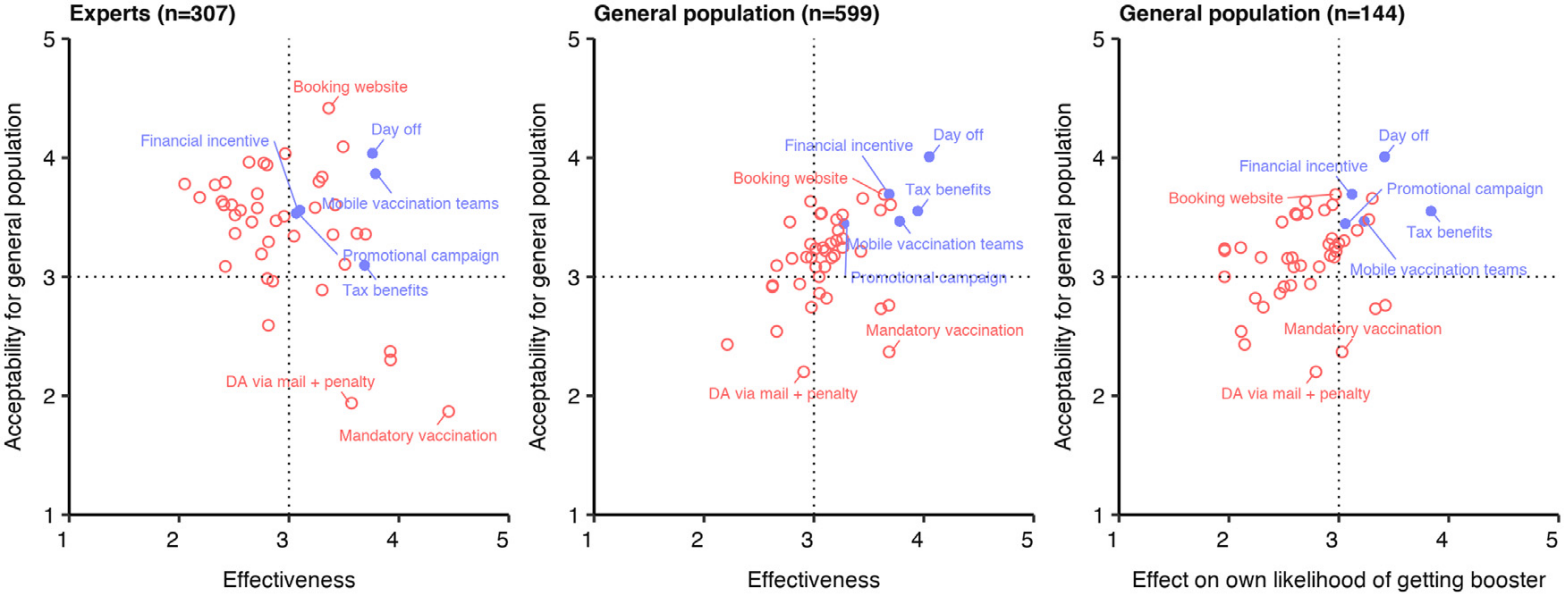
Relationship between expected effectiveness and acceptability of interventions as rated by experts and respondents from the general population. Blue circles indicate interventions with mean ratings above the midpoint of the scale (>3, scale: 1–5) on both effectiveness and acceptability (upper right quadrant) for all samples. DA: Default appointment.

Figure 3 goes into greater detail and displays all interventions with regard to both their expected effectiveness and acceptability, as judged by experts or respondents from the general population. While the experts expected that the introduction of vaccination mandates and different sanctions (e.g., restricted access to public spaces for people who have not received the booster vaccination) would be most effective in increasing COVID-19 booster uptake, respondents from the general population rated incentives such as a day off work after getting vaccinated or financial incentives as most effective in increasing overall and own booster uptake. Importantly, mandatory vaccination received the lowest and the second-lowest acceptability rating by experts and respondents from the general population, respectively. Acceptability was evaluated highest by experts for a website to book appointments for booster vaccination (third place by respondents from the general population), whereas a day off work after vaccination received the highest rating by respondents from the general population, both for themselves and the expected overall acceptability to the general population (third place by experts).

Several reasons might cause (mis)alignment in evaluations between experts and respondents from the general population,^22^ therefore, we aimed to identify interventions that received positive evaluations across these two groups. To this end, we identified interventions that received mean ratings above the midpoint of the response scale (>3 on a scale from 1–5; the upper right quadrants in Figure 3) across respondent groups and evaluation criteria. Overall, 16 interventions had positive evaluations on both effectiveness and acceptability by experts, and 26 in evaluations by the general population (9 when referring to intentions of own booster uptake). Taken together, 5 out of all 46 interventions were rated positively by both experts and the general population regarding effectiveness and acceptability (Figure 3, blue dots). These interventions are: (1) a day off work after getting vaccinated, (2) financial incentives (either lottery or fixed payment), (3) tax benefits (e.g., reduction of health insurance rate), (4) promotional campaigns (e.g., stressing who else can indirectly benefit from their own booster vaccination, such as vulnerable persons or healthcare personnel), and (5) mobile vaccination teams (e.g., allowing people to get vaccinated at their private and work places).

## Discussion

Using a novel crowdsourcing approach in relation to vaccination policies and interventions, we present insights on relevant decision criteria to implement interventions aimed at increasing uptake of COVID-19 booster vaccines in eligible adults from the general population. In times where governments grapple with strategies to increase booster vaccine uptake in pandemic-fatigued populations, but large-scale comparative evidence on the effectiveness and acceptability of such strategies is still missing, intervention ideas and subjective evaluations by a large number of experts and respondents from the general population may well provide useful insights to help governmental and non-governmental institutions choose from a range of interventions. Thus, although our crowdsourcing approach applies various elements of well-known crowdsourcing methods in the health domain, it does not include an objective evaluation of the proposed interventions due to a lack of data for such a large and heterogenous set of interventions.

The results indicate that, in view of the diversity of criteria for evaluation, there is no single best intervention or intervention class to promote COVID-19 booster vaccine uptake, especially when expert and general population evaluations are both taken into account. In particular, some interventions that are deemed effective are deemed less acceptable (e.g., mandates) and may elicit counter behaviors such as active opposition. Such detrimental social effects of coercive measures—which could undermine the effectiveness of an intervention aimed to increase COVID-19 booster vaccination uptake—have been reported also with regard to other vaccinations, including COVID-19 primary vaccination.^23^, ^24^, ^25^, ^26^

We also find that, not surprisingly, evaluations of experts and of people from the general population do not always align. Most noteworthy, experts saw a substantially smaller (positive) association between an intervention's acceptability and its effectiveness compared to respondents from the general population. Such misalignment in perceptions between these groups could have serious consequences if an intervention is recommended by experts—and eventually implemented—that is deemed effective but less accepted by the general population. On the positive side, we can identify several interventions that are evaluated positively with regard to both anticipated effectiveness and acceptability by both experts and respondents from the general population. Interestingly, three of these five interventions rely on incentives, i.e., providing some kind of positive reward for booster vaccination. For COVID-19 primary vaccination, there is mixed evidence regarding the effectiveness of (monetary) incentives, with some studies claiming that even small financial benefits could increase vaccine uptake,^27^^,^^28^ whereas others showing that only large incentives of several hundred or even thousand euros would suffice to increase vaccine uptake.^29^, ^30^, ^31^ Importantly, the effectiveness of incentives likely depends on the specific population it aims to target. For instance, when people are uncertain or feel psychological and structural constraints (e.g., time, stress), financial incentives could make a difference, whereas people who are not getting vaccinated because of strong negative sentiments regarding vaccination would probably be less sensitive to such incentives. Regarding COVID-19 booster vaccination, the target population already decided in favor of COVID-19 primary vaccination in the past and should therefore be more positive toward vaccination compared to unvaccinated people. Indeed, our results are in line with recent experimental evidence showing that financial incentives are more likely to increase intentions for booster vaccination than for primary vaccination.^12^ Whether the proposed interventions would also be able to increase primary vaccine uptake was not the focus of the present study. Nevertheless, we provide additional data on the potential effectiveness in of the proposed interventions to also increase COVID-19 primary vaccination (see Figure 2—all interventions had very small expected effects). We call for further research on potentially different levels of effectiveness and acceptability of incentives for different vaccines and target groups.

Our study has several limitations. First, despite the large number of experts, their evaluations could still be subject to systematic bias. It is important to note that most of the experts had a disciplinary background in economics, psychology, or public health. Further, in the second-phase survey we provided financial incentives via a lottery payment scheme, which could have attracted expert participants who are more positive toward (financial) incentives, potentially affecting their own evaluations of such interventions. Other biases could potentially occur based on the methodological background and expertise of the experts, which we have not assessed. Regarding the respondents from the general population, we relied on online samples from the UK and the US. Naturally, this sampling procedure could lead to some bias toward interventions that rely on online information (although we do not see strong evidence for this in our data) and findings from both populations cannot directly be transferred to other countries, particularly with diverging cultural backgrounds. Second, and related to the first limitation, the feasibility of the interventions in a specific context and country still requires scrutiny. Some interventions are linked to specific institutional regulations (e.g., tax benefits) or require operational systems that may not be feasible in every setting (e.g., providing financial incentives to vaccinated persons). Moreover, our data provides insights on several evaluation criteria that could be weighted differently by different institutions. We particularly focused on effectiveness and acceptability as these are arguably crucial criteria for selecting interventions and, additionally, because we had evaluations by both experts and people from the general population for both of these criteria. Third, we should emphasize (again) that our study aimed to provide insights on potentially useful interventions to increase COVID-19 booster vaccine uptake. As such, we do not claim that the same interventions would also apply or receive similar evaluations when targeted to other vaccines. Yet, it would be interesting to investigate whether similar effects (e.g., on the evaluation of different intervention classes but also on the association between different intervention criteria and differences thereof between different evaluator groups) do also replicate for other vaccines. Finally, our crowdsourcing approach was very minimal—lacking elements of lay community engagement (in generating new intervention proposals) or competition between proposals (due to the absence of objective evaluation criteria)^17^^,^^32^—which appeared most appropriate and feasible in the present context. Taken together, these limitations suggest that before implementing any of these interventions, they should be adapted, contextualized, and tested for actual effectiveness in order to be applied in different settings. Importantly, however, subjective evaluations of interventions should not replace objective evaluation efforts (e.g., by conducting randomized controlled trials assessing vaccine uptake), but should be seen as a first step to select interventions for further evaluation and potential implementation.

In conclusion, the present overview of established and novel interventions, along with their evaluations by more than 300 experts and about 600 people from the UK and US general population, is a useful resource for governmental and non-governmental institutions which seek to select and implement interventions that can be used to increase COVID-19 booster vaccine uptake, now and in the near future. Additionally, in the absence of any large-scale comparative evidence on the usefulness of behavioral interventions, the proposed multi-phase crowdsourcing approach could serve as a blueprint for similar situations.

## Contributors

Conceptualization: R.B., M.K., C.B.; Methodology: R.B., M.K., C.B., Y.L., P.S., N.B., G.C., J.L., G.L., M.S., C.S.; Investigation: Y.L., P.S., R.B., M.K., C.B.; Visualization: P.S., Y.L.; Funding acquisition: R.B., M.K.; Project administration: R.B.; Supervision: R.B.; Writing – original draft: R.B.; Writing – review & editing: R.B., M.K., C.B., Y.L., P.S., N.B., G.C., J.L., G.L., M.S., C.S.

## Data sharing statement

Anonymized data and analyses scripts as well as supplementary information and survey materials are publicly available via the Open Science Framework (https://osf.io/ab54u/).

## Declaration of interests

R.B., C.B., Y.L., G.C., J.L., M.S., and C.S. have nothing to disclose. P.S. reports personal fees from WHO outside the submitted work. N.B. reports personal fees from WHO, US CDC, Merck, and Novartis outside the submitted work. J.L. reports grants from WHO, UNICEF, and US CDC, as well as personal fees from Therapeutic Goods Administration outside the submitted work. G.L. reports non-financial support for Level2, as well as personal fees from Florida Blue and Highmark Health outside the submitted work. M.K. reports grants from Austrian Science Fund (grant no.: SFB F63) related to the submitted work.

## Appendix Supplementary materials

Image, application 1
