# Supplementary material for: Crowdsourcing interventions to promote uptake of COVID-19 booster vaccines
Source: eClinicalMedicine. 2022 Sep 5;53:101632. doi: 10.1016/j.eclinm.2022.101632 (PMC9444232; doi:10.1016/j.eclinm.2022.101632)

SUPPLEMTARY MATERIAL:

Crowdsourcing Interventions to Promote Uptake of COVID-19 Booster Vaccines

Robert Böhm[1,2,3] *, Cornelia Betsch[4,5,6], Yana Litovsky[7], Philipp Sprengholz[4,6], Noel T. Brewer[8 +], Gretchen Chapman[9 +], Julie Leask[10 +], George Loewenstein[9 +], Martha Scherzer[11 +], Cass R. Sunstein[12 +], Michael Kirchler[7]

[1] Faculty of Psychology, University of Vienna, Austria

[2] Department of Psychology, University of Copenhagen; 1354 Copenhagen K, Denmark

[3] Copenhagen Center for Social Data Science (SODAS), University of Copenhagen, Denmark

[4] Media and Communication Science, University of Erfurt, Germany

[5] Center for Empirical Research in Economics and Behavioral Sciences (CEREB), University of Erfurt, Germany

[6] Bernhard Nocht Institute for Tropical Medicine (BNITM), Hamburg, Germany

[7] Department of Banking and Finance, University of Innsbruck, Austria

[8] Department of Health Behavior, Gillings School of Global Public Health and Lineberger Comprehensive Cancer Center, University of North Carolina at Chapel Hill, USA

[9] Department of Social and Decision Sciences, Carnegie Mellon University, USA

[10] Faculty of Medicine and Health, The University of Sydney, Australia

[11] Consultant, Behavioral and Cultural Insights Unit, WHO Regional Office for Europe, Copenhagen, Denmark

[12] Harvard Law School, Harvard University, USA

* Correspondence to: Robert Böhm, Faculty of Psychology, University of Vienna, Universitätsstrasse 7, 1010 Vienna, Austria. Email: robert.boehm@univie.ac.at

[+] These authors are ordered alphabetically.

Contents

	Page
Unique Interventions proposed by experts (Table S1)	3
Regression analyses: Expert ratings (Tables S2-S10)	11
Regression analyses: General population ratings (Tables S11-S16)	29
Mean values of expert ratings (Figure S1)	41
Mean values of general population rations (Figure S2)	42
Correlations between expert evaluation criteria (Figure S3)	43
Correlations between general population evaluation criteria (Figure S4)	44
Mean values (from top to bottom) across evaluation criteria: Expert ratings (Figure S5-S13)	45
Mean values (from top to bottom) across evaluation criteria: General population ratings (Figure S14-S19)	54

Table S1. Unique interventions proposed by experts.

#	Short name	Description	Classification							
			1	2	3	4	5	6	7	8
1	Vaccination festival	Widely advertised public social event with music, games, prizes, and vaccination stations. Vaccinated individuals will receive vouchers for games and other social activities (e.g., entry to a haunted house). Additional information stations with medical staff to distribute information and discuss COVID-19 booster vaccination.	x		x		x	x		
2	Free party/concert	Large social event with famous DJs/music bands, food trucks, etc. Access is granted (for free) to all people who got the booster vaccination. People could also get access when getting the booster vaccination at the entrance.			x		x	x	x	
3	Norm letter	Send weekly letters about the neighborhood's uptake rate of booster vaccination. Happy smiley if the letter recipient has already received the booster vaccination, sad smiley if s/he has not.			x	x		x		
4	Norms by time	Provide information about the share of people vaccinated in the same period (e.g., July 2020) who have already received the booster vaccination.			x	x				
5	Social media campaign	Sharing information about the benefits of booster vaccination on social media platforms (including collaboration features provided at some platforms, e.g. Instagram). Recruitment of influencers to join the campaign and spread the word.	x	x	x					

			1	2	3	4	5	6	7	8
6	Day off	People receive a paid day off after booster vaccination. This would be paid by the state.						x		
7	Restricted access	Access to public places (e.g., restaurants, airplanes, etc.) or work places will only be granted to boostered people. Vaccination status will be checked by responsible persons (e.g., owners of shops or restaurants) via official vaccination documents.							x	
8	Local role models	Recruit local role models (e.g., community leaders, local celebrities) to promote booster vaccination.		x	x					
9	Letter from doctors	Send letter from local general practitioner to inform about the value of booster vaccination.	x							
10	Mobile vaccination teams	Send mobile vaccination teams to allow people get their booster vaccination at private and work places (e.g., gym, shopping centers, company).					x			
11	Health professionals' calls	Health professionals call people who have not yet received a booster vaccination to let them know that an appointment has been scheduled for them. They provide further information and answer questions if requested. After the appointment has been scheduled, people will also receive a reminder text message 24 hours prior to the appointment.	x			x				
12	Targeted phone calls	Communities use citizen register to call persons over 60 by telephone and arrange a booster vaccination appointment if they are willing to have one.				x				

			1	2	3	4	5	6	7	8
13	Mandatory vaccination	Mandate booster vaccination with financial penalties, monitored and executed by the responsible health authority.								x
14	Booking website	Website that centralizes all available locations to get booster vaccinations and allows efficient booking of vaccination appointments (e.g., minimizing waiting and travel time for people).				x				
15	Motivational interviewing	General practitioners should ask every patient about their vaccination status. If they have not yet received the booster vaccine, they apply motivational interviewing, a patient-centered, directive approach to counseling with the goal of building intrinsic motivation to change behavior.	x	x		x				
16	DA via app	Signing up eligible people automatically for booster appointments. Appointments are shared by push notification and can be rescheduled (both time and location) via a central app. In case the recipient wants to decline the invitation, s/he will be offered to reschedule the appointment instead and receives additional information about individual and collective benefits of the booster vaccination.	x			x				
17	DA via mail	Signing up eligible people automatically for booster appointments. Appointments are shared via regular mail. Cancelation requires to explain the reasons and listen to explanation on safety/benefits of booster vaccination.	x			x				

			1	2	3	4	5	6	7	8
18	DA via mail + penalty	Signing up eligible people automatically for booster appointments. Appointments are shared via regular mail. Rescheduling or cancelation of appointment via phone. Failure to show up at appointment results in a small to medium-sized fine (e.g., 50€). Cancelation requires to explain the reasons and listen to explanation on safety/benefits of booster vaccination.	x			x				x
19	DA via mail + transportation	Signing up eligible people automatically for booster appointments. Appointments are shared via regular mail. Offer transportation to appointment to those without other transportation options. Cancelation requires to explain the reasons and listen to explanation on safety/benefits of booster vaccination.	x			x	x			
20	Vaccination stations	Easily accessible stations where people can get information about booster vaccination. In case there is already a network of testing stations, information and vaccines should be made available there too.	x				x			
21	Financial incentive	Boostered people enter a lottery for a large prize (e.g., 10.000 €) or will receive a certain but smaller prize (e.g., 5 €).						x		
22	Lottery + referral	Boostered people receive a lottery ticket for a large prize (e.g., monthly income reward for life). People receive additional lottery tickets if they recommend the booster to others, who then get vaccinated (referral), so lottery tickets increase the more others can be motivated to get vaccinated.		x				x		

			1	2	3	4	5	6	7	8
23	Decision aid + health promotion	Simplified summary of the evidence-based and legal consequences of (not) getting the booster vaccine. This includes the consequences for oneself and for others. For instance, when getting the booster vaccination (versus not getting it) you are X times less likely to develop symptoms and Y times less likely to be hospitalized if you get infected. It also includes a list of things that you will be (not) able to do with (without) the booster vaccination, such as attending a wedding party or visiting a restaurant (depending on the country's regulations). A summary of evidence includes a list of endorsers for the booster vaccination, from scientists, politicians, religious leaders, etc.	x	x	x					
24	Necessity campaign	Increase knowledge about the necessity of booster vaccination (e.g., benefits of vaccination, danger of COVID-19) via mass media, such as TV and magazine ads.	x	x						
25	Media legislation	Legislation that all media has to report responsibly and truthfully regarding the pandemic.	x							
26	Promotional campaign	Mass and social media campaign stressing who else (in addition to oneself) can be protected or helped by getting the booster vaccine, including personal stories of vulnerable persons (e.g., older persons, immunocomprised persons) or healthcare personnel (e.g., intensive care nurses). Communication also via direct communication (e.g., doctors, trusted community leaders).	x	x	x					

			1	2	3	4	5	6	7	8
27	Personalized calculator	Public website that allows people to enter which vaccine/s they have received and when, whether they have had confirmed or suspected COVID-19 and their symptoms, their age, potential vaccine-adverse events experienced with earlier COVID-19 vaccinations, etc. The website would then provide personalized information about when to receive the booster vaccination and which vaccines are recommended.	x			x				
28	Reservation	Eligible persons are informed that a valuable vaccine dose worth X€ has been reserved for them and may be wasted if they do not claim it within a given period of time.		x		x				
29	Insurance sanction	Health insurance premium rises by X€ if booster vaccination cannot be proven within a certain time frame.								x
30	Reciprocity appeal	Mass media advertisement with young person who lost his/her grandparent during the pandemic and who is now getting the booster vaccination to protect the viewer's older loved ones, asking whether you will do the same. Includes a descriptive norm message on how many others (plan to) do the same.		x	x					
31	Free snacks	Give free snacks (e.g. burgers) after people get the booster vaccination.						x		
32	Information support for media	Website for media/journalists with up-to-date reliable information on benefits & risks of vaccination and disease (including new variants) in non-technical jargon. Information should be provided and regularly updated by scientific experts from universities.	x							

			1	2	3	4	5	6	7	8
33	Stories from suffering people	Share stories about people from the local community (e.g., people living in the same city) who describe the severe course of their COVID-19 infection after not having received the (booster) vaccination.		x	x					
34	Relative risks for hospitalization	Hospitalization rates of vaccinated versus unvaccinated people should be communicated for different population and risk groups (e.g., people over 80 years of age) to allow relative risk calculation for the own group.	x	x						
35	Help others to educate	Provide information for people about how to educate others (e.g., family members, friends) about the value of booster vaccination.	x	x	x					
36	Personalized text message	People receive personalized text message (addressing them by their name) highlighting the benefits of getting the booster vaccination.	x	x						
37	Tax benefits	Finanical benefits for boostered people, e.g., tax benefits, reduction of health insurance rate.						x		
38	Information website	Website with basic information on the benefits of booster vaccination, where and how to get it, using easy language and graphical illustrations.	x							
39	Mind-changing stories	Short videos on social media platforms of persons who explain their initial concerns before getting the booster vaccine, and their reasoning for why they eventually decided to get it. Stories should include facts but also personal reasons.	x	x	x					

			1	2	3	4	5	6	7	8
40	Q&A session	People can submit questions and concerns regarding booster vaccinations. Experts will create written and video answers which are provided online and in public Q&A events.	x							
41	Awareness ads	Short videos with famous comedians without lengthy explanations to be streamed on TV.			x					
42	Punch card	Card indicates that 2 out of 3 necessary vaccinations have already been completed, but one is still missing to achieve the protection goal.				x				
43	Mass vaccination	Weekly vaccination events (weekly at the same day and the same location) where people can go and get the booster vaccine without prior appointment.					x			
44	Donation	For every booster vaccination, there will be one dose donated to a developing country.						x		
45	Documentary	Documentary for non-experts to explain the benefits and necessity of booster vaccination, explained by trusted health experts from various disciplines. Documentary should be shown on public TV.	x	x	x					
46	Implementation intentions	Provide prompt to people that helps them plan their booster vaccination (e.g., in newspapers, flyer in supermarkets to fill in; "If X happens, I will make an appointment for booster vaccination.")				x				

Note. 1: Education. 2: Persuasion. 3: Modeling. 4: Psychological enablement. 5: Environmental restructuring. 6: Incentivization. 7: Restriction. 8: Sanction. DA: Default appointment. For definitions of classification criteria, see Table S2, top panel.

We are grateful to all the experts who proposed and evaluated interventions. Below is a list of experts who proposed interventions in the phase-one survey and agreed to be named (in alphabetical order): M. Alston, H. Alves, C. Anderson, N. I. A. Aziz, L. Balafoutas, B. Balmford, J. Baron, M. H. Birnbaum, J. E. Bogard, C. Bonner, G. Briscese, K. Brix, A. Calero Valdez, P. Conway, A. K. Erenel, M. P. Espinosa, J. Fehr, K. Gangl, O. Genschow, A. Gantman, A. Gagneux-Brunon, H. Ghirll, W. Göhner, M. R. P. Grossmann, K. Hein-Peters, M. Heller, G. Hertel, S. Heuse, A. K. Huemer, A. R. Homar, M. Janneck, P. Jiménez, L. Keller, G. Kleiter, C. König-Kersting, S. Koupidis, S. Kurzenhäuser-Carstens, J. Laferton, H. Lanthorn, G. Lämmlin, A. Lieberoth, S. Lippke, F. Löschmann, L. Lucas, D. B. Mak, E. Matthies, G. Mayraz, B. C. McCannon, F. Neunhoeffer, A.-M. Nussberger, E. Pattenden, S. Palan, C. Pitterle, T. Rothmund, H. Rusch, B. Rüschoff, K. Sachse, A. Sadrieh, J. B. Schmutz, A. Strobel, E. M. VanEpps, P. Wallace, K. Westhoff, L. Windsteiger, A. Zabala, B. Zikmund-Fisher, G. Zilles

Table S2. Expert ratings: Effect on affordability.

	Base model			Extended model		
Predictors	*B*	*95 % CI*	*p*	*B*	*95 % CI*	*p*
(Intercept)	2.81	2.69 – 2.93	**<0.001**	2.28	1.44 – 3.12	**<0.001**
Education	0.11	0.01 – 0.20	**0.037**	0.11	0.00 – 0.21	**0.047**
Persuasion	0.01	-0.11 – 0.13	0.866	-0.02	-0.15 – 0.12	0.808
Modeling	-0.32	-0.43 – -0.21	**<0.001**	-0.29	-0.41 – -0.17	**<0.001**
Psychological enablement	0.04	-0.06 – 0.15	0.417	0.02	-0.10 – 0.13	0.770
Environmental restructuring	0.66	0.52 – 0.80	**<0.001**	0.67	0.52 – 0.82	**<0.001**
Incentivization	0.78	0.65 – 0.91	**<0.001**	0.77	0.64 – 0.91	**<0.001**
Restriction	0.13	-0.10 – 0.36	0.276	0.12	-0.12 – 0.36	0.330
Sanction	0.40	0.22 – 0.59	**<0.001**	0.35	0.15 – 0.55	**0.001**
Age				0.00	-0.01 – 0.02	0.645
Gender: Male (baseline: female)				-0.11	-0.26 – 0.04	0.139
Gender: Non-binary (baseline: female)				0.08	-0.72 – 0.89	0.842
Gender: Prefer not to say (baseline: female)				-0.24	-1.39 – 0.92	0.687
Profession: Healthcare provider (baseline: scientist)				0.45	-0.28 – 1.18	0.226
Profession: Other practitioner (baseline: scientist)				0.30	-0.23 – 0.82	0.269
Profession: Other (baseline: scientist)				0.11	-0.10 – 0.33	0.307
Education: Economics (baseline: medicine or healthcare)				0.38	-0.34 – 1.10	0.297
Education: Public health (baseline: medicine or healthcare)				0.22	-0.50 – 0.95	0.546

Education: Psychology (baseline: medicine or healthcare)				0.47	-0.23 – 1.18	0.191
Education: Other (baseline: medicine or healthcare)				0.33	-0.39 – 1.06	0.369
Participation in the first survey (baseline: no participation)				0.01	-0.17 – 0.20	0.904
Working experience (years)				0.00	-0.02 – 0.02	0.822
**Random Effects**						
σ^2	1.24			1.26		
τ_{00}	0.19_{ID}			0.19_{ID}		
ICC	0.13_{ID}			0.13_{ID}		
Observations	2619			2362		
Marginal R^2 / Conditional R^2	0.110 / 0.227			0.117 / 0.235		

Note: Results from mixed effects regressions with a random effect of participant ID.

Table S3. Expert ratings: Effect on practicability.

	Base model			Extended model		
Predictors	*B*	*95 % CI*	*p*	*B*	*95 % CI*	*p*
(Intercept)	4.04	3.93 – 4.15	**<0.001**	4.19	3.39 – 5.00	**<0.001**
Education	-0.42	-0.51 – -0.33	**<0.001**	-0.41	-0.51 – -0.32	**<0.001**
Persuasion	0.02	-0.10 – 0.13	0.780	0.02	-0.10 – 0.14	0.707
Modeling	-0.03	-0.13 – 0.07	0.604	-0.04	-0.15 – 0.06	0.421
Psychological enablement	-0.22	-0.32 – -0.12	**<0.001**	-0.20	-0.30 – -0.10	**<0.001**
Environmental restructuring	-0.10	-0.22 – 0.03	0.137	-0.10	-0.23 – 0.03	0.143
Incentivization	-0.39	-0.50 – -0.27	**<0.001**	-0.38	-0.50 – -0.26	**<0.001**
Restriction	-0.52	-0.73 – -0.32	**<0.001**	-0.53	-0.75 – -0.31	**<0.001**
Sanction	-0.66	-0.83 – -0.49	**<0.001**	-0.65	-0.83 – -0.47	**<0.001**
Age				-0.00	-0.02 – 0.01	0.663
Gender: Male (baseline: female)				-0.10	-0.24 – 0.04	0.170
Gender: Non-binary (baseline: female)				-0.57	-1.34 – 0.20	0.148
Gender: Prefer not to say (baseline: female)				-0.68	-1.79 – 0.43	0.230
Profession: Healthcare provider (baseline: scientist)				0.05	-0.65 – 0.74	0.894
Profession: Other practitioner (baseline: scientist)				0.45	-0.05 – 0.96	0.078
Profession: Other (baseline: scientist)				0.02	-0.18 – 0.23	0.829
Education: Economics (baseline: medicine or healthcare)				0.05	-0.64 – 0.73	0.892
Education: Public health (baseline: medicine or healthcare)				-0.26	-0.96 – 0.43	0.457

Education: Psychology (baseline: medicine or healthcare)				-0.03	-0.71 – 0.64	0.927
Education: Other (baseline: medicine or healthcare)				0.06	-0.63 – 0.76	0.856
Participation in the first survey (baseline: no participation)				-0.16	-0.33 – 0.02	0.081
Working experience (years)				0.01	-0.01 – 0.02	0.556
**Random Effects**						
σ^2	0.99			1.00		
τ_{00}	0.20 $_{ID}$			0.19 $_{ID}$		
ICC	0.17 $_{ID}$			0.16 $_{ID}$		
Observations	2615			2360		
Marginal R^2 / Conditional R^2	0.055 / 0.211			0.065 / 0.217		

Note: Results from mixed effects regressions with a random effect of participant ID.

Table S4. Expert ratings: Effect on effectiveness.

	Base model			Extended model		
Predictors	*B*	*95 % CI*	*p*	*B*	*95 % CI*	*p*
(Intercept)	3.01	2.89 – 3.12	**<0.001**	3.40	2.63 – 4.18	**<0.001**
Education	-0.26	-0.35 – -0.17	**<0.001**	-0.26	-0.36 – -0.17	**<0.001**
Persuasion	0.26	0.14 – 0.38	**<0.001**	0.27	0.14 – 0.39	**<0.001**
Modeling	-0.37	-0.47 – -0.26	**<0.001**	-0.37	-0.48 – -0.25	**<0.001**
Psychological enablement	0.14	0.04 – 0.24	**0.008**	0.15	0.04 – 0.25	**0.007**
Environmental restructuring	0.41	0.27 – 0.54	**<0.001**	0.41	0.27 – 0.55	**<0.001**
Incentivization	0.01	-0.11 – 0.13	0.825	0.00	-0.12 – 0.13	0.950
Restriction	0.25	0.03 – 0.46	**0.026**	0.23	0.01 – 0.46	**0.045**
Sanction	0.97	0.79 – 1.14	**<0.001**	0.97	0.78 – 1.16	**<0.001**
Age				0.00	-0.01 – 0.02	0.959
Gender: Male (baseline: female)				-0.29	-0.43 – -0.16	**<0.001**
Gender: Non-binary (baseline: female)				-0.66	-1.40 – 0.08	0.079
Gender: Prefer not to say (baseline: female)				-0.34	-1.40 – 0.73	0.535
Profession: Healthcare provider (baseline: scientist)				0.23	-0.44 – 0.90	0.502
Profession: Other practitioner (baseline: scientist)				-0.05	-0.53 – 0.43	0.839
Profession: Other (baseline: scientist)				0.23	0.03 – 0.43	**0.026**
Education: Economics (baseline: medicine or healthcare)				-0.25	-0.91 – 0.41	0.451
Education: Public health (baseline: medicine or healthcare)				-0.49	-1.16 – 0.17	0.146

Education: Psychology (baseline: medicine or healthcare)				-0.30	-0.95 – 0.35	0.366
Education: Other (baseline: medicine or healthcare)				-0.24	-0.91 – 0.43	0.490
Participation in the first survey (baseline: no participation)				-0.05	-0.22 – 0.12	0.548
Working experience (years)				-0.00	-0.02 – 0.02	0.964
**Random Effects**						
σ^2	1.10			1.11		
τ_{00}	0.18 $_{ID}$			0.16 $_{ID}$		
ICC	0.14 $_{ID}$			0.13 $_{ID}$		
Observations	2617			2362		
Marginal R^2 / Conditional R^2	0.078 / 0.206			0.104 / 0.217		

Note: Results from mixed effects regressions with a random effect of participant ID.

Table S5. Expert ratings: Effect on acceptability for stakeholders.

	Base model			Extended model		
Predictors	*B*	*95 % CI*	*p*	*B*	*95 % CI*	*p*
(Intercept)	3.74	3.63 – 3.85	**<0.001**	4.39	3.66 – 5.13	**<0.001**
Education	-0.20	-0.29 – -0.11	**<0.001**	-0.18	-0.28 – -0.08	**<0.001**
Persuasion	0.02	-0.10 – 0.13	0.772	0.06	-0.07 – 0.18	0.361
Modeling	0.22	0.11 – 0.32	**<0.001**	0.20	0.09 – 0.31	**<0.001**
Psychological enablement	-0.09	-0.19 – 0.01	0.070	-0.07	-0.17 – 0.04	0.217
Environmental restructuring	0.15	0.02 – 0.28	**0.028**	0.13	-0.01 – 0.26	0.068
Incentivization	-0.67	-0.79 – -0.55	**<0.001**	-0.61	-0.74 – -0.49	**<0.001**
Restriction	-1.02	-1.24 – -0.81	**<0.001**	-1.03	-1.26 – -0.81	**<0.001**
Sanction	-1.37	-1.55 – -1.20	**<0.001**	-1.36	-1.54 – -1.17	**<0.001**
Age				-0.00	-0.01 – 0.01	0.959
Gender: Male (baseline: female)				0.04	-0.09 – 0.17	0.536
Gender: Non-binary (baseline: female)				-0.20	-0.89 – 0.50	0.578
Gender: Prefer not to say (baseline: female)				-0.68	-1.68 – 0.33	0.185
Profession: Healthcare provider (baseline: scientist)				-0.50	-1.13 – 0.14	0.125
Profession: Other practitioner (baseline: scientist)				0.17	-0.28 – 0.62	0.457
Profession: Other (baseline: scientist)				-0.09	-0.28 – 0.10	0.353
Education: Economics (baseline: medicine or healthcare)				-0.64	-1.26 – -0.01	**0.045**
Education: Public health (baseline: medicine or healthcare)				-0.75	-1.38 – -0.12	**0.020**

Education: Psychology (baseline: medicine or healthcare)				-0.64	-1.26 – -0.03	**0.039**
Education: Other (baseline: medicine or healthcare)				-0.64	-1.27 – -0.00	**0.048**
Participation in the first survey (baseline: no participation)				0.01	-0.15 – 0.17	0.940
Working experience (years)				-0.00	-0.02 – 0.01	0.778
**Random Effects**						
σ^2	1.09			1.08		
τ_{00}	0.13_{ID}			0.13_{ID}		
ICC	0.11_{ID}			0.11_{ID}		
Observations	2610			2353		
Marginal R^2 / Conditional R^2	0.146 / 0.238			0.152 / 0.244		

Note: Results from mixed effects regressions with a random effect of participant ID.

Table S6. Expert ratings: Effect on acceptability for general population.

	Base model			Extended model		
Predictors	*B*	*95 % CI*	*p*	*B*	*95 % CI*	*p*
(Intercept)	3.77	3.66 – 3.88	**<0.001**	4.13	3.41 – 4.85	**<0.001**
Education	-0.27	-0.36 – -0.18	**<0.001**	-0.27	-0.37 – -0.18	**<0.001**
Persuasion	0.03	-0.08 – 0.15	0.555	0.06	-0.07 – 0.18	0.367
Modeling	-0.06	-0.16 – 0.05	0.272	-0.07	-0.18 – 0.04	0.202
Psychological enablement	-0.14	-0.24 – -0.04	**0.005**	-0.12	-0.22 – -0.01	**0.026**
Environmental restructuring	0.27	0.14 – 0.40	**<0.001**	0.25	0.11 – 0.38	**<0.001**
Incentivization	-0.21	-0.33 – -0.09	**0.001**	-0.20	-0.32 – -0.07	**0.002**
Restriction	-1.09	-1.30 – -0.88	**<0.001**	-1.09	-1.31 – -0.87	**<0.001**
Sanction	-1.60	-1.77 – -1.43	**<0.001**	-1.58	-1.76 – -1.40	**<0.001**
Age				0.00	-0.01 – 0.01	0.959
Gender: Male (baseline: female)				-0.12	-0.25 – 0.01	0.065
Gender: Non-binary (baseline: female)				-0.04	-0.73 – 0.64	0.901
Gender: Prefer not to say (baseline: female)				-0.08	-1.06 – 0.91	0.881
Profession: Healthcare provider (baseline: scientist)				-0.17	-0.79 – 0.45	0.583
Profession: Other practitioner (baseline: scientist)				0.19	-0.26 – 0.65	0.406
Profession: Other (baseline: scientist)				-0.20	-0.39 – -0.02	**0.034**
Education: Economics (baseline: medicine or healthcare)				-0.20	-0.82 – 0.41	0.513
Education: Public health (baseline: medicine or healthcare)				-0.26	-0.88 – 0.36	0.413

Education: Psychology (baseline: medicine or healthcare)		-0.32	-0.92 – 0.28	0.302
Education: Other (baseline: medicine or healthcare)		-0.23	-0.85 – 0.39	0.474
Participation in the first survey (baseline: no participation)		-0.04	-0.20 – 0.12	0.622
Working experience (years)		-0.00	-0.02 – 0.01	0.732
**Random Effects**				
σ^2	1.03	1.04		
τ_{00}	0.15 $_{ID}$	0.13 $_{ID}$		
ICC	0.12 $_{ID}$	0.11 $_{ID}$		
Observations	2604	2352		
Marginal R^2 / Conditional R^2	0.144 / 0.251	0.150 / 0.243		

Note: Results from mixed effects regressions with a random effect of participant ID.

Table S7. Expert ratings: Effect on probability of non-pharmaceutical side effects.

	Base model			Extended model		
Predictors	*B*	*95 % CI*	*p*	*B*	*95 % CI*	*p*
(Intercept)	2.19	2.08 – 2.31	**<0.001**	1.89	0.92 – 2.86	**<0.001**
Education	0.07	-0.02 – 0.16	0.107	0.07	-0.02 – 0.17	0.142
Persuasion	0.10	-0.01 – 0.21	0.082	0.07	-0.05 – 0.19	0.244
Modeling	0.07	-0.03 – 0.17	0.173	0.10	-0.01 – 0.21	0.068
Psychological enablement	0.08	-0.02 – 0.17	0.121	0.03	-0.07 – 0.13	0.559
Environmental restructuring	0.05	-0.07 – 0.18	0.416	0.02	-0.11 – 0.15	0.775
Incentivization	0.55	0.43 – 0.66	**<0.001**	0.54	0.42 – 0.66	**<0.001**
Restriction	0.95	0.74 – 1.16	**<0.001**	0.95	0.73 – 1.17	**<0.001**
Sanction	1.37	1.20 – 1.54	**<0.001**	1.32	1.14 – 1.50	**<0.001**
Age				-0.01	-0.03 – 0.01	0.367
Gender: Male (baseline: female)				-0.06	-0.23 – 0.12	0.524
Gender: Non-binary (baseline: female)				-0.07	-1.00 – 0.87	0.888
Gender: Prefer not to say (baseline: female)				0.06	-1.28 – 1.40	0.931
Profession: Healthcare provider (baseline: scientist)				0.35	-0.50 – 1.19	0.421
Profession: Other practitioner (baseline: scientist)				-0.07	-0.68 – 0.54	0.817
Profession: Other (baseline: scientist)				0.17	-0.08 – 0.42	0.178
Education: Economics (baseline: medicine or healthcare)				0.55	-0.28 – 1.39	0.193
Education: Public health (baseline: medicine or healthcare)				0.56	-0.28 – 1.40	0.191

Education: Psychology (baseline: medicine or healthcare)				0.67	-0.15 – 1.49	0.108
Education: Other (baseline: medicine or healthcare)				0.71	-0.14 – 1.55	0.101
Participation in the first survey (baseline: no participation)				0.17	-0.05 – 0.38	0.130
Working experience (years)				0.00	-0.02 – 0.03	0.686
**Random Effects**						
σ^2	0.99			1.01		
τ_{00}	0.32_{ID}			0.33_{ID}		
ICC	0.25_{ID}			0.25_{ID}		
Observations	2610			2359		
Marginal R^2 / Conditional R^2	0.114 / 0.332			0.125 / 0.339		

Note: Results from mixed effects regressions with a random effect of participant ID.

Table S8. Expert ratings: Effect on inequity.

	Base model			Extended model		
Predictors	*B*	*95 % CI*	*p*	*B*	*95 % CI*	*p*
(Intercept)	2.74	2.65 – 2.83	**<0.001**	2.71	2.13 – 3.29	**<0.001**
Education	0.13	0.06 – 0.21	**0.001**	0.12	0.04 – 0.20	**0.004**
Persuasion	-0.02	-0.11 – 0.08	0.714	-0.06	-0.16 – 0.04	0.225
Modeling	0.09	0.01 – 0.17	**0.036**	0.13	0.04 – 0.21	**0.005**
Psychological enablement	0.07	-0.01 – 0.15	0.093	0.04	-0.05 – 0.12	0.379
Environmental restructuring	-0.29	-0.39 – -0.18	**<0.001**	-0.32	-0.43 – -0.21	**<0.001**
Incentivization	0.12	0.02 – 0.22	**0.015**	0.10	-0.01 – 0.20	0.067
Restriction	0.75	0.57 – 0.92	**<0.001**	0.75	0.57 – 0.93	**<0.001**
Sanction	0.41	0.27 – 0.56	**<0.001**	0.37	0.22 – 0.52	**<0.001**
Age				-0.00	-0.02 – 0.01	0.435
Gender: Male (baseline: female)				-0.05	-0.16 – 0.05	0.298
Gender: Non-binary (baseline: female)				0.01	-0.54 – 0.56	0.969
Gender: Prefer not to say (baseline: female)				0.16	-0.63 – 0.95	0.695
Profession: Healthcare provider (baseline: scientist)				-0.06	-0.56 – 0.44	0.806
Profession: Other practitioner (baseline: scientist)				0.11	-0.25 – 0.47	0.559
Profession: Other (baseline: scientist)				-0.02	-0.17 – 0.13	0.820
Education: Economics (baseline: medicine or healthcare)				0.15	-0.34 – 0.64	0.545
Education: Public health (baseline: medicine or healthcare)				0.36	-0.14 – 0.85	0.158

Education: Psychology (baseline: medicine or healthcare)				0.21	-0.28 – 0.69	0.403
Education: Other (baseline: medicine or healthcare)				0.25	-0.25 – 0.75	0.328
Participation in the first survey (baseline: no participation)				0.05	-0.08 – 0.17	0.479
Working experience (years)				0.01	-0.01 – 0.02	0.436
**Random Effects**						
σ^2	0.70			0.71		
τ_{00}	0.08_{ID}			0.08_{ID}		
ICC	0.10_{ID}			0.10_{ID}		
Observations	2598			2350		
Marginal R^2 / Conditional R^2	0.045 / 0.144			0.054 / 0.149		

Note: Results from mixed effects regressions with a random effect of participant ID.

Table S9. Expert ratings: Effect on universality across countries.

	Base model			Extended model		
Predictors	*B*	*95 % CI*	*p*	*B*	*95 % CI*	*p*
(Intercept)	3.33	3.20 – 3.45	**<0.001**	3.96	2.96 – 4.97	**<0.001**
Education	-0.25	-0.34 – -0.15	**<0.001**	-0.24	-0.34 – -0.13	**<0.001**
Persuasion	0.16	0.04 – 0.28	**0.007**	0.17	0.04 – 0.29	**0.010**
Modeling	0.04	-0.07 – 0.15	0.504	0.05	-0.06 – 0.17	0.372
Psychological enablement	-0.23	-0.33 – -0.12	**<0.001**	-0.21	-0.32 – -0.10	**<0.001**
Environmental restructuring	0.12	-0.01 – 0.26	0.075	0.14	-0.01 – 0.28	0.062
Incentivization	-0.60	-0.72 – -0.47	**<0.001**	-0.60	-0.73 – -0.47	**<0.001**
Restriction	-0.32	-0.54 – -0.10	**0.005**	-0.38	-0.62 – -0.15	**0.001**
Sanction	-0.93	-1.11 – -0.74	**<0.001**	-0.90	-1.09 – -0.70	**<0.001**
Age				-0.01	-0.03 – 0.01	0.266
Gender: Male (baseline: female)				0.06	-0.12 – 0.23	0.544
Gender: Non-binary (baseline: female)				-0.35	-1.32 – 0.61	0.475
Gender: Prefer not to say (baseline: female)				-1.03	-2.42 – 0.35	0.144
Profession: Healthcare provider (baseline: scientist)				-0.21	-1.09 – 0.66	0.630
Profession: Other practitioner (baseline: scientist)				-0.04	-0.67 – 0.59	0.896
Profession: Other (baseline: scientist)				-0.05	-0.31 – 0.21	0.730
Education: Economics (baseline: medicine or healthcare)				-0.35	-1.21 – 0.51	0.422
Education: Public health (baseline: medicine or healthcare)				-0.54	-1.41 – 0.33	0.220

Education: Psychology (baseline: medicine or healthcare)				-0.43	-1.27 – 0.42	0.323
Education: Other (baseline: medicine or healthcare)				-0.30	-1.17 – 0.58	0.506
Participation in the first survey (baseline: no participation)				-0.02	-0.24 – 0.21	0.886
Working experience (years)				0.01	-0.01 – 0.03	0.296
**Random Effects**						
σ^2	1.14			1.17		
τ_{00}	0.35 $_{ID}$			0.34 $_{ID}$		
ICC	0.24 $_{ID}$			0.23 $_{ID}$		
Observations	2615			2363		
Marginal R^2 / Conditional R^2	0.070 / 0.290			0.078 / 0.287		

Note: Results from mixed effects regressions with a random effect of participant ID.

Table S10. Expert ratings: Effect on previously unvaccinated people.

	Base model			Extended model		
Predictors	*B*	*95 % CI*	*p*	*B*	*95 % CI*	*p*
(Intercept)	3.48	3.39 – 3.56	**<0.001**	3.70	3.06 – 4.33	**<0.001**
Education	-0.20	-0.27 – -0.14	**<0.001**	-0.20	-0.27 – -0.13	**<0.001**
Persuasion	0.13	0.05 – 0.21	**0.003**	0.13	0.04 – 0.22	**0.003**
Modeling	-0.13	-0.21 – -0.06	**0.001**	-0.14	-0.22 – -0.06	**0.001**
Psychological enablement	-0.17	-0.25 – -0.10	**<0.001**	-0.18	-0.26 – -0.10	**<0.001**
Environmental restructuring	0.16	0.07 – 0.26	**0.001**	0.17	0.07 – 0.27	**0.001**
Incentivization	-0.12	-0.20 – -0.03	**0.009**	-0.12	-0.21 – -0.03	**0.011**
Restriction	0.05	-0.11 – 0.20	0.541	0.03	-0.14 – 0.19	0.749
Sanction	0.04	-0.09 – 0.17	0.536	0.08	-0.06 – 0.21	0.266
Age				-0.01	-0.02 – 0.01	0.344
Gender: Male (baseline: female)				-0.08	-0.20 – 0.03	0.148
Gender: Non-binary (baseline: female)				0.04	-0.56 – 0.65	0.885
Gender: Prefer not to say (baseline: female)				-0.51	-1.38 – 0.36	0.250
Profession: Healthcare provider (baseline: scientist)				0.33	-0.22 – 0.88	0.238
Profession: Other practitioner (baseline: scientist)				0.04	-0.36 – 0.45	0.829
Profession: Other (baseline: scientist)				0.20	0.03 – 0.36	**0.019**
Education: Economics (baseline: medicine or healthcare)				-0.07	-0.61 – 0.47	0.801
Education: Public health (baseline: medicine or healthcare)				-0.12	-0.67 – 0.43	0.670

Education: Psychology (baseline: medicine or healthcare)		-0.14	-0.67 – 0.40	0.615
Education: Other (baseline: medicine or healthcare)		-0.20	-0.75 – 0.35	0.474
Participation in the first survey (baseline: no participation)		-0.05	-0.19 – 0.09	0.478
Working experience (years)		0.01	-0.00 – 0.02	0.174
**Random Effects**				
σ^2	0.55	0.56		
τ_{00}	0.14 $_{\text{ID}}$	0.13 $_{\text{ID}}$		
ICC	0.20 $_{\text{ID}}$	0.18 $_{\text{ID}}$		
Observations	2582	2333		
Marginal R^2 / Conditional R^2	0.027 / 0.218	0.049 / 0.223		

Note: Results from mixed effects regressions with a random effect of participant ID.

Table S11. General population ratings: Effect on booster uptake.

	**Base model**			**Extended model**		
Predictors	*B*	*95 % CI*	*p*	*B*	*95 % CI*	*p*
(Intercept)	3.43	3.35 – 3.51	**<0.001**	3.39	3.09 – 3.70	**<0.001**
Education	-0.17	-0.22 – -0.11	**<0.001**	-0.17	-0.22 – -0.11	**<0.001**
Persuasion	0.08	0.00 – 0.15	**0.038**	0.08	0.00 – 0.15	**0.036**
Modeling	-0.28	-0.34 – -0.22	**<0.001**	-0.28	-0.34 – -0.22	**<0.001**
Psychological enablement	-0.44	-0.50 – -0.38	**<0.001**	-0.44	-0.50 – -0.38	**<0.001**
Environmental restructuring	0.16	0.08 – 0.24	**<0.001**	0.16	0.08 – 0.24	**<0.001**
Incentivization	-0.04	-0.12 – 0.03	0.243	-0.05	-0.12 – 0.03	0.211
Restriction	0.00	-0.13 – 0.13	0.988	-0.00	-0.13 – 0.13	0.994
Sanction	0.16	0.05 – 0.27	**0.003**	0.16	0.05 – 0.27	**0.004**
Age				-0.00	-0.01 – 0.00	0.067
Gender: male (baseline: female)				-0.12	-0.23 – -0.01	**0.032**
Country: US (baseline: UK)				-0.21	-0.32 – -0.10	**<0.001**
Education: less than high school (baseline: high school or equivalent)				-0.03	-0.64 – 0.58	0.921
Education: post-graduate education (baseline: high school or equivalent)				-0.10	-0.26 – 0.05	0.198
Education: some college (baseline: high school or equivalent)				-0.10	-0.26 – 0.05	0.187
Vaccinated (baseline: unvaccinated)				0.38	0.23 – 0.54	**<0.001**
Liberal political orientation				0.02	-0.01 – 0.06	0.224

**Random Effects**		
σ^2	0.90	0.90
τ_{00}	0.40 $_{ID}$	0.37 $_{ID}$
ICC	0.31 $_{ID}$	0.29 $_{ID}$
Observations	5990	5980
Marginal R^2 / Conditional R^2	0.042 / 0.336	0.072 / 0.340

Note: Results from mixed effects regressions with a random effect of participant ID.

Table S12. General population ratings: Effect on own booster intention.

	Base model			Extended model		
Predictors	*B*	*95 % CI*	*p*	*B*	*95 % CI*	*p*
(Intercept)	3.02	2.80 – 3.24	**<0.001**	3.12	2.23 – 4.01	**<0.001**
Education	-0.14	-0.28 – -0.01	**0.032**	-0.15	-0.28 – -0.02	**0.029**
Persuasion	0.07	-0.10 – 0.24	0.445	0.07	-0.10 – 0.24	0.440
Modeling	-0.32	-0.47 – -0.17	**<0.001**	-0.32	-0.47 – -0.17	**<0.001**
Psychological enablement	-0.37	-0.51 – -0.23	**<0.001**	-0.37	-0.52 – -0.23	**<0.001**
Environmental restructuring	-0.10	-0.29 – 0.09	0.294	-0.10	-0.29 – 0.09	0.284
Incentivization	-0.02	-0.20 – 0.15	0.813	-0.02	-0.20 – 0.15	0.796
Restriction	0.32	0.00 – 0.64	**0.048**	0.32	0.00 – 0.63	**0.048**
Sanction	0.15	-0.10 – 0.40	0.239	0.15	-0.10 – 0.39	0.247
Age				-0.01	-0.02 – 0.00	0.071
Gender: male (baseline: female)				-0.09	-0.44 – 0.25	0.591
Country: US (baseline: UK)				0.20	-0.18 – 0.57	0.305
Education: less than high school (baseline: high school or equivalent)				-1.75	-3.15 – -0.35	**0.014**
Education: post-graduate education (baseline: high school or equivalent)				-0.18	-0.69 – 0.33	0.481
Education: some college (baseline: high school or equivalent)				-0.35	-0.83 – 0.12	0.144
Liberal political orientation				0.11	0.01 – 0.21	**0.031**

**Random Effects**		
σ^2	1.02	1.02
τ_{00}	0.87 $_{ID}$	0.80 $_{ID}$
ICC	0.46 $_{ID}$	0.44 $_{ID}$
Observations	1240	1240
Marginal R^2 / Conditional R^2	0.027 / 0.477	0.085 / 0.489

Note: Results from mixed effects regressions with a random effect of participant ID. Subsample of participants who have not yet received a booster vaccine at the time of the study (n=144).

Table S13. General population ratings: Effect on perceived coercion.

	**Base model**			**Extended model**		
Predictors	*B*	*95 % CI*	*p*	*B*	*95 % CI*	*p*
(Intercept)	2.21	2.12 – 2.30	**<0.001**	3.22	2.87 – 3.56	**<0.001**
Education	0.01	-0.05 – 0.08	0.691	0.01	-0.05 – 0.08	0.674
Persuasion	0.46	0.38 – 0.54	**<0.001**	0.45	0.37 – 0.53	**<0.001**
Modeling	-0.16	-0.24 – -0.09	**<0.001**	-0.16	-0.23 – -0.09	**<0.001**
Psychological enablement	0.50	0.44 – 0.57	**<0.001**	0.50	0.43 – 0.57	**<0.001**
Environmental restructuring	0.04	-0.05 – 0.13	0.421	0.04	-0.05 – 0.13	0.422
Incentivization	0.45	0.36 – 0.53	**<0.001**	0.44	0.36 – 0.53	**<0.001**
Restriction	1.04	0.90 – 1.19	**<0.001**	1.04	0.89 – 1.19	**<0.001**
Sanction	1.76	1.63 – 1.88	**<0.001**	1.75	1.63 – 1.88	**<0.001**
Age				-0.00	-0.01 – 0.00	0.127
Gender: male (baseline: female)				-0.23	-0.35 – -0.11	**<0.001**
Country: US (baseline: UK)				-0.22	-0.35 – -0.09	**0.001**
Education: less than high school (baseline: high school or equivalent)				-0.10	-0.79 – 0.59	0.777
Education: post-graduate education (baseline: high school or equivalent)				-0.09	-0.27 – 0.09	0.318
Education: some college (baseline: high school or equivalent)				0.05	-0.12 – 0.23	0.554
Vaccinated (baseline: unvaccinated)				-0.37	-0.55 – -0.19	**<0.001**
Liberal political orientation				-0.06	-0.10 – -0.02	**0.002**

**Random Effects**		
σ^2	1.13	1.13
τ_{00}	0.53 $_{ID}$	0.48 $_{ID}$
ICC	0.32 $_{ID}$	0.30 $_{ID}$
Observations	5990	5980
Marginal R^2 / Conditional R^2	0.121 / 0.402	0.153 / 0.404

Note: Results from mixed effects regressions with a random effect of participant ID.

Table S14. General population ratings: Effect on reactance.

	Base model			Extended model		
Predictors	*B*	*95 % CI*	*p*	*B*	*95 % CI*	*p*
(Intercept)	1.43	1.35 – 1.52	**<0.001**	2.99	2.66 – 3.31	**<0.001**
Education	0.13	0.08 – 0.18	**<0.001**	0.13	0.08 – 0.18	**<0.001**
Persuasion	0.18	0.12 – 0.25	**<0.001**	0.18	0.11 – 0.24	**<0.001**
Modeling	-0.05	-0.10 – 0.01	0.090	-0.05	-0.10 – 0.01	0.092
Psychological enablement	0.59	0.54 – 0.64	**<0.001**	0.59	0.53 – 0.64	**<0.001**
Environmental restructuring	0.02	-0.05 – 0.09	0.569	0.02	-0.05 – 0.09	0.585
Incentivization	0.26	0.19 – 0.32	**<0.001**	0.25	0.19 – 0.32	**<0.001**
Restriction	0.91	0.80 – 1.03	**<0.001**	0.91	0.80 – 1.03	**<0.001**
Sanction	1.52	1.43 – 1.62	**<0.001**	1.52	1.43 – 1.62	**<0.001**
Age				-0.00	-0.01 – 0.00	0.159
Gender: male (baseline: female)				-0.15	-0.27 – -0.03	**0.013**
Country: US (baseline: UK)				-0.07	-0.19 – 0.06	0.290
Education: less than high school (baseline: high school or equivalent)				0.27	-0.39 – 0.93	0.423
Education: post-graduate education (baseline: high school or equivalent)				0.08	-0.09 – 0.25	0.371
Education: some college (baseline: high school or equivalent)				0.08	-0.09 – 0.25	0.340
Vaccinated (baseline: unvaccinated)				-0.78	-0.95 – -0.61	**<0.001**
Liberal political orientation				-0.15	-0.19 – -0.11	**<0.001**

**Random Effects**		
σ^2	0.71	0.71
τ_{00}	0.61 $_{ID}$	0.46 $_{ID}$
ICC	0.46 $_{ID}$	0.40 $_{ID}$
Observations	5990	5980
Marginal R^2 / Conditional R^2	0.129 / 0.532	0.230 / 0.535

Note: Results from mixed effects regressions with a random effect of participant ID. Reactance was measured with four items about how angry, frustrated, disturbed participants felt about the respective intervention and how much they perceived it as a restriction of their freedom.

Table S15. General population ratings: Effect on acceptability for general population.

	Base model			Extended model		
Predictors	*B*	*95 % CI*	*p*	*B*	*95 % CI*	*p*
(Intercept)	3.53	3.44 – 3.61	**<0.001**	3.31	2.99 – 3.62	**<0.001**
Education	-0.12	-0.18 – -0.06	**<0.001**	-0.12	-0.17 – -0.06	**<0.001**
Persuasion	-0.04	-0.11 – 0.04	0.333	-0.04	-0.11 – 0.04	0.333
Modeling	-0.16	-0.23 – -0.10	**<0.001**	-0.16	-0.23 – -0.10	**<0.001**
Psychological enablement	-0.44	-0.50 – -0.38	**<0.001**	-0.44	-0.50 – -0.38	**<0.001**
Environmental restructuring	0.05	-0.03 – 0.13	0.210	0.05	-0.03 – 0.13	0.218
Incentivization	0.01	-0.07 – 0.08	0.856	0.00	-0.07 – 0.08	0.896
Restriction	-0.51	-0.65 – -0.38	**<0.001**	-0.51	-0.64 – -0.37	**<0.001**
Sanction	-0.97	-1.08 – -0.86	**<0.001**	-0.97	-1.08 – -0.86	**<0.001**
Age				0.00	-0.00 – 0.01	0.137
Gender: male (baseline: female)				-0.03	-0.14 – 0.09	0.648
Country: US (baseline: UK)				-0.08	-0.19 – 0.04	0.198
Education: less than high school (baseline: high school or equivalent)				0.01	-0.62 – 0.64	0.982
Education: post-graduate education (baseline: high school or equivalent)				-0.07	-0.23 – 0.09	0.387
Education: some college (baseline: high school or equivalent)				-0.07	-0.23 – 0.09	0.379
Vaccinated (baseline: unvaccinated)				0.12	-0.05 – 0.28	0.166
Liberal political orientation				0.02	-0.01 – 0.06	0.249

**Random Effects**		
σ^2	0.92	0.92
τ_{00}	0.40 $_{ID}$	0.40 $_{ID}$
ICC	0.30 $_{ID}$	0.30 $_{ID}$
Observations	5990	5980
Marginal R^2 / Conditional R^2	0.067 / 0.349	0.072 / 0.352

Note: Results from mixed effects regressions with a random effect of participant ID.

Table S16. General population ratings: Effect on activism intentions.

	Base model			Extended model		
Predictors	*B*	*95 % CI*	*p*	*B*	*95 % CI*	*p*
(Intercept)	1.28	1.22 – 1.34	**<0.001**	2.52	2.25 – 2.79	**<0.001**
Education	0.04	0.01 – 0.08	**0.015**	0.04	0.01 – 0.07	**0.016**
Persuasion	0.03	-0.01 – 0.08	0.123	0.03	-0.01 – 0.07	0.137
Modeling	-0.03	-0.07 – 0.00	0.071	-0.03	-0.07 – 0.00	0.068
Psychological enablement	0.18	0.15 – 0.22	**<0.001**	0.18	0.15 – 0.22	**<0.001**
Environmental restructuring	-0.02	-0.07 – 0.03	0.388	-0.02	-0.07 – 0.03	0.380
Incentivization	0.06	0.01 – 0.10	**0.010**	0.06	0.01 – 0.10	**0.010**
Restriction	0.37	0.29 – 0.45	**<0.001**	0.37	0.29 – 0.45	**<0.001**
Sanction	0.72	0.66 – 0.79	**<0.001**	0.72	0.66 – 0.79	**<0.001**
Age				-0.00	-0.01 – -0.00	**0.002**
Gender: male (baseline: female)				-0.07	-0.16 – 0.03	0.189
Country: US (baseline: UK)				0.02	-0.08 – 0.12	0.751
Education: less than high school (baseline: high school or equivalent)				0.17	-0.38 – 0.72	0.538
Education: post-graduate education (baseline: high school or equivalent)				-0.02	-0.16 – 0.12	0.809
Education: some college (baseline: high school or equivalent)				-0.02	-0.16 – 0.12	0.760
Vaccinated (baseline: unvaccinated)				-0.57	-0.71 – -0.42	**<0.001**
Liberal political orientation				-0.10	-0.13 – -0.07	**<0.001**

**Random Effects**		
σ^2	0.31	0.31
τ_{00}	0.42 $_{ID}$	0.34 $_{ID}$
ICC	0.57 $_{ID}$	0.52 $_{ID}$
Observations	5990	5980
Marginal R^2 / Conditional R^2	0.051 / 0.592	0.154 / 0.594

Note: Results from mixed effects regressions with a random effect of participant ID. Activism intentions were measured with four items (signing a petition, joining a demonstration, joining a lawsuit, and mobilizing others to fight the respective intervention).

Figure S1. Mean values of expert ratings.

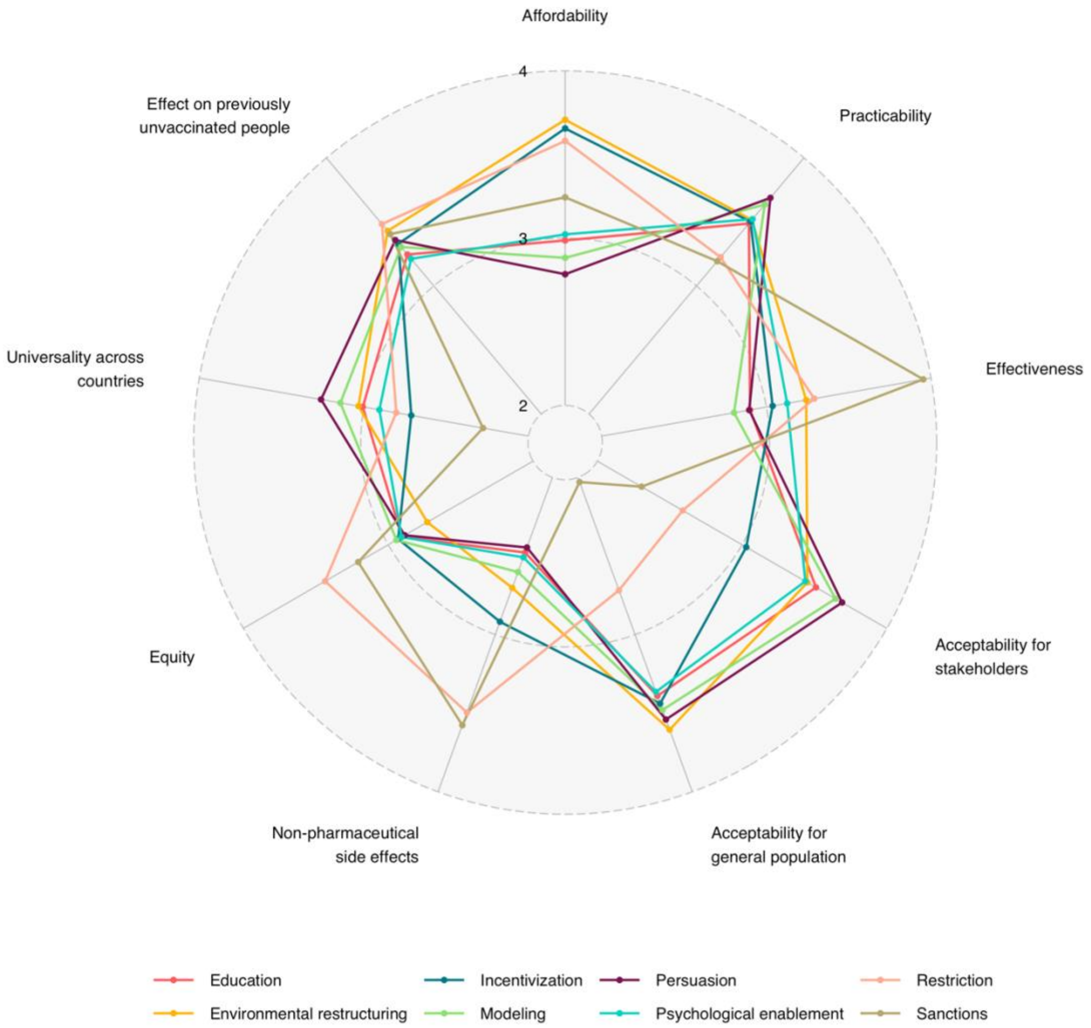

Figure S2. Mean values of general population ratings.

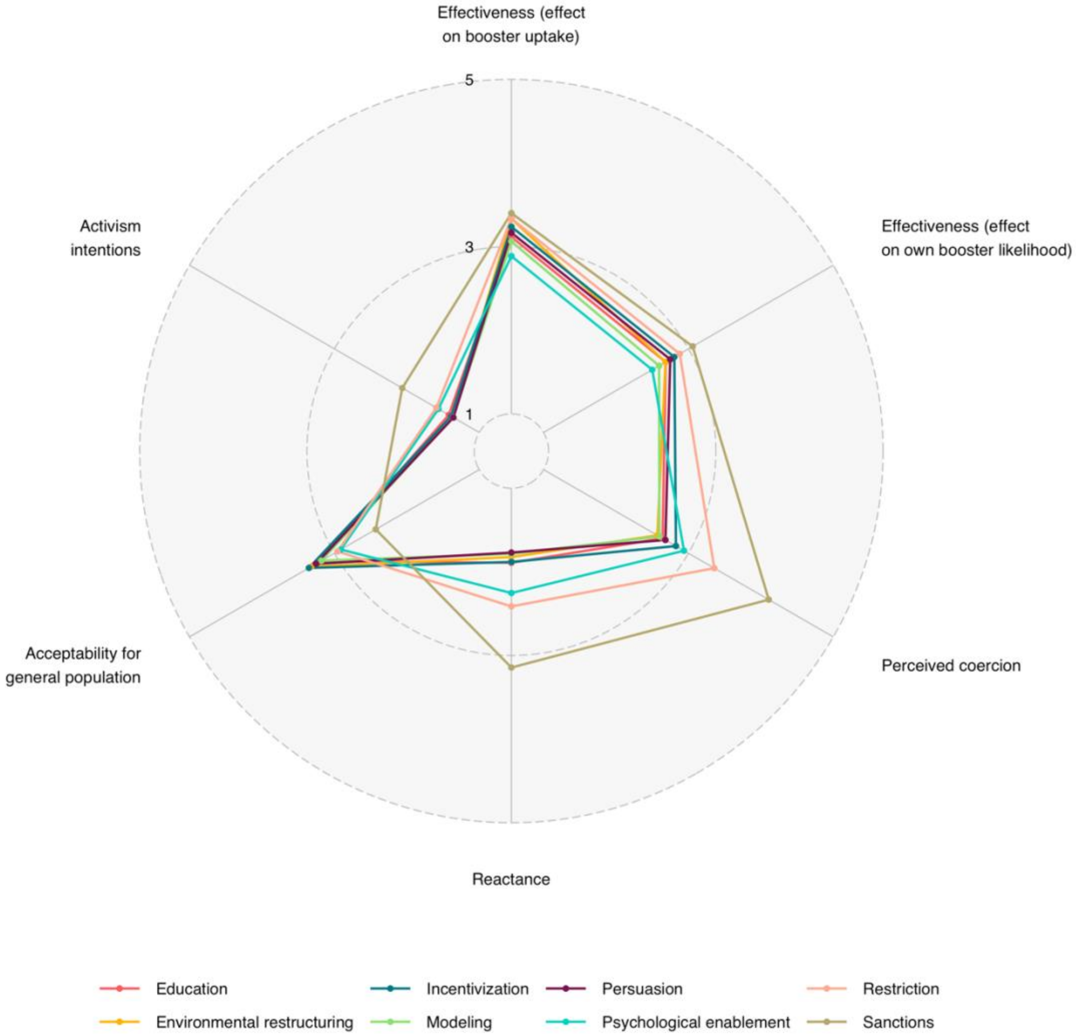

Figure S3. Correlations between expert evaluation criteria (across all interventions).

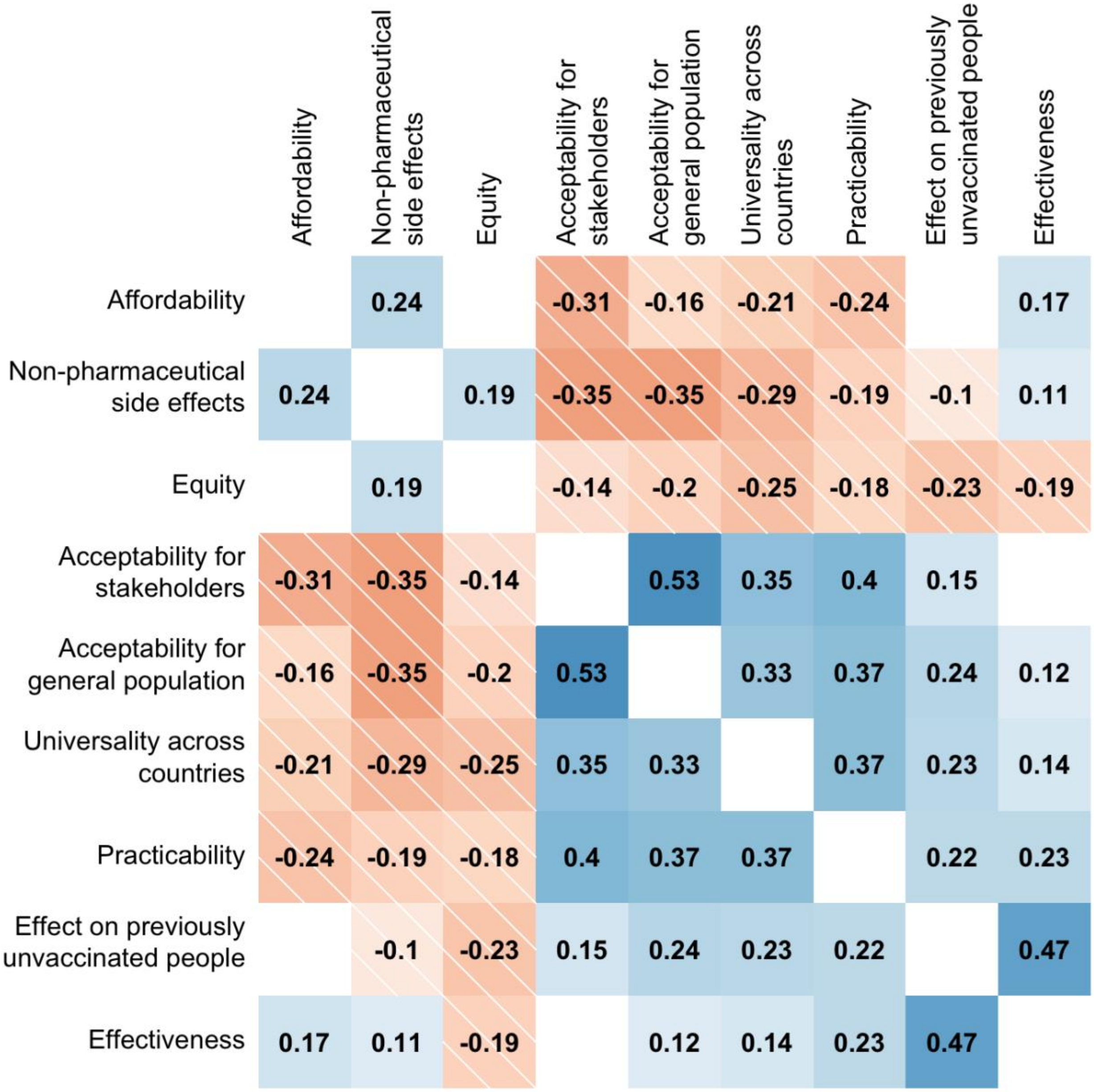

Figure S4. Correlations between general population evaluation criteria (across all interventions).

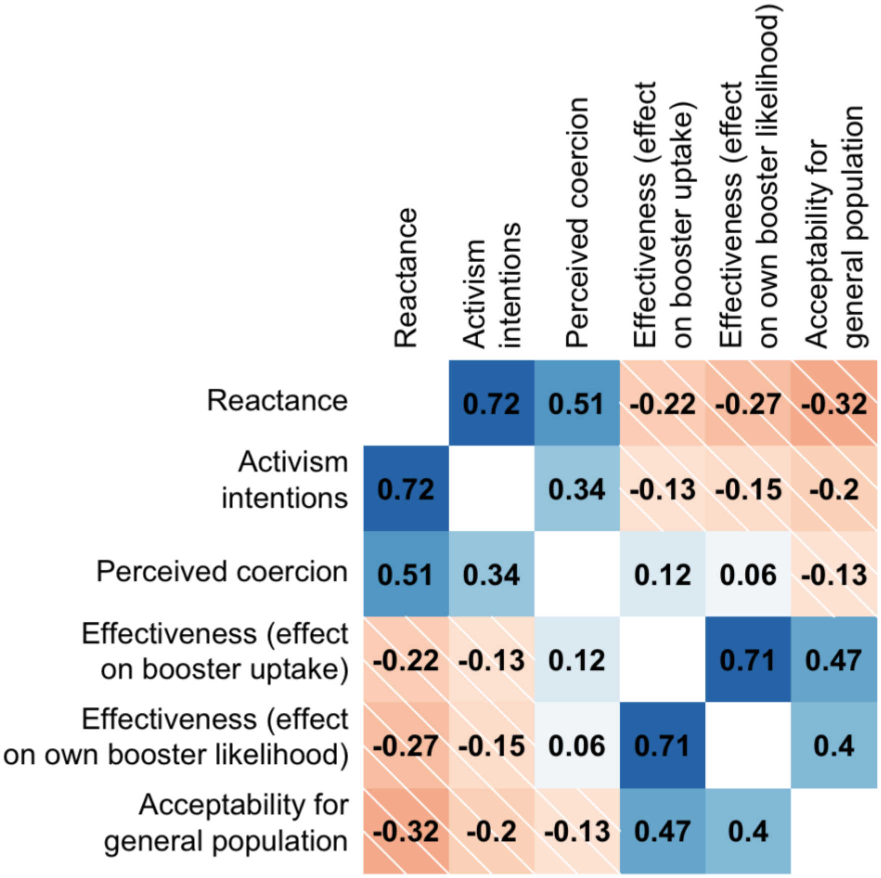

Figure S5. Expert ratings: Mean values in affordability.

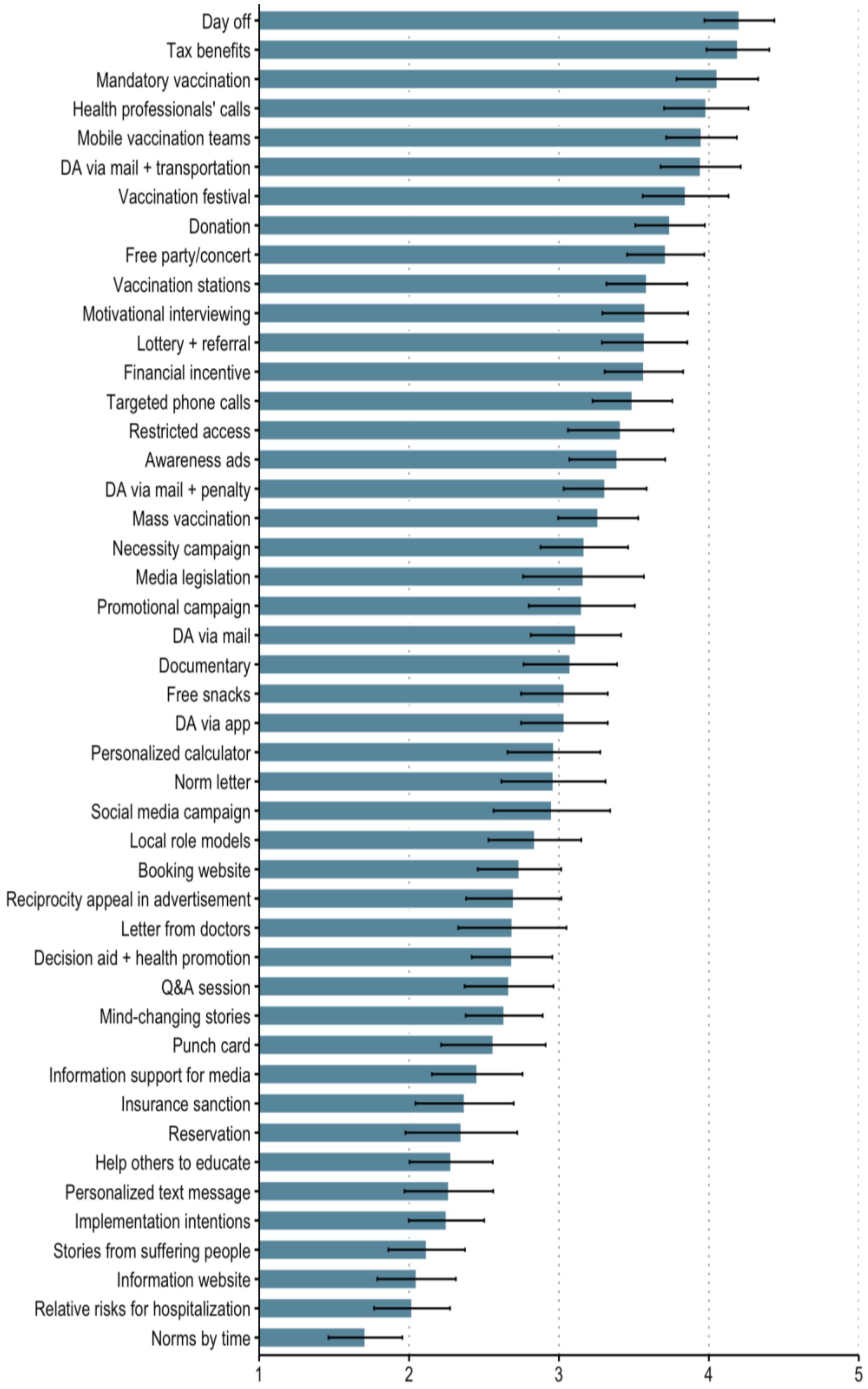

Figure S6. Expert ratings: Mean values in practicability.

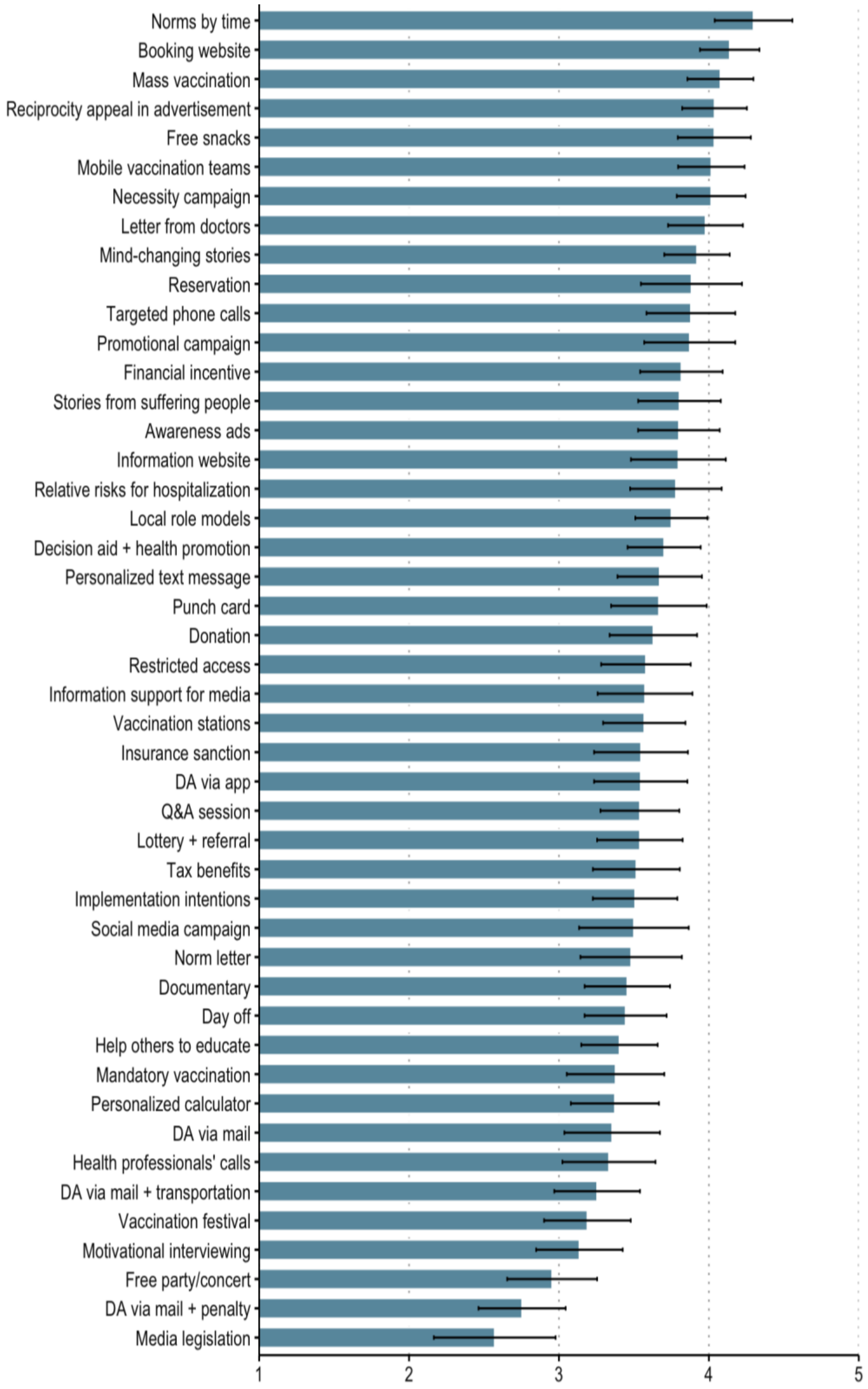

Figure S7. Expert ratings: Mean values in effectiveness.

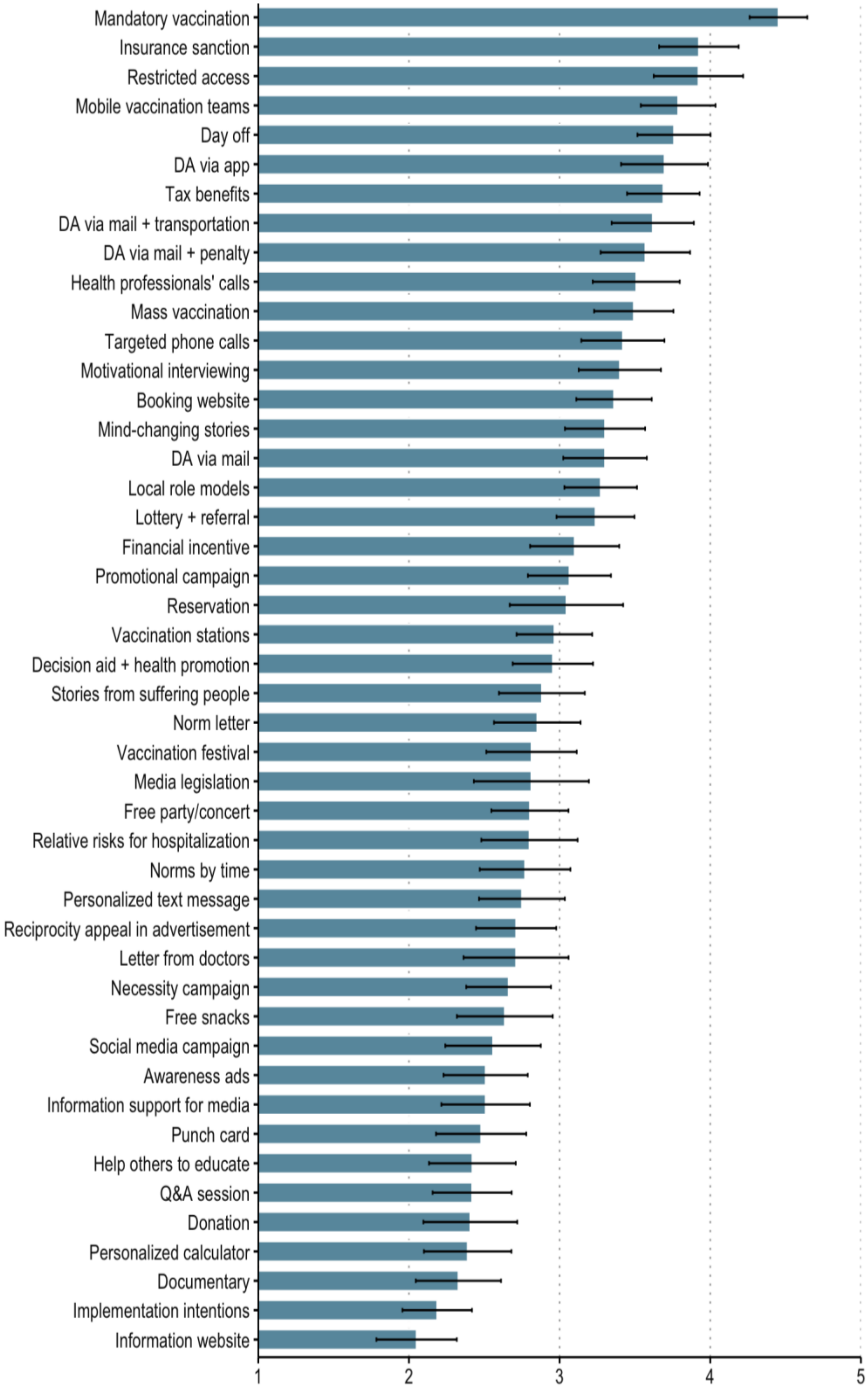

Figure S8. Expert ratings: Mean values in acceptability for stakeholders.

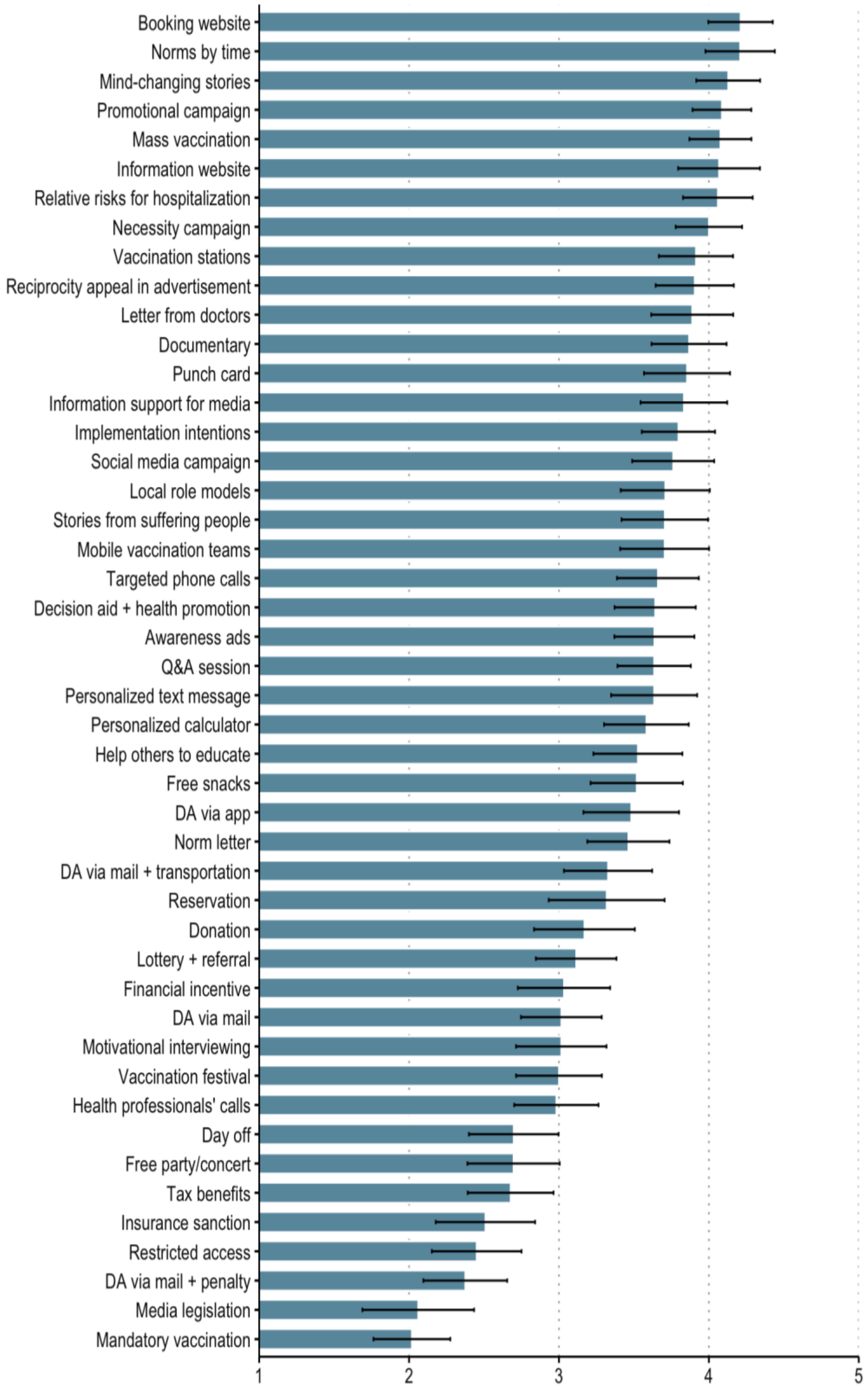

Figure S9. Expert ratings: Mean values in acceptability for general population.

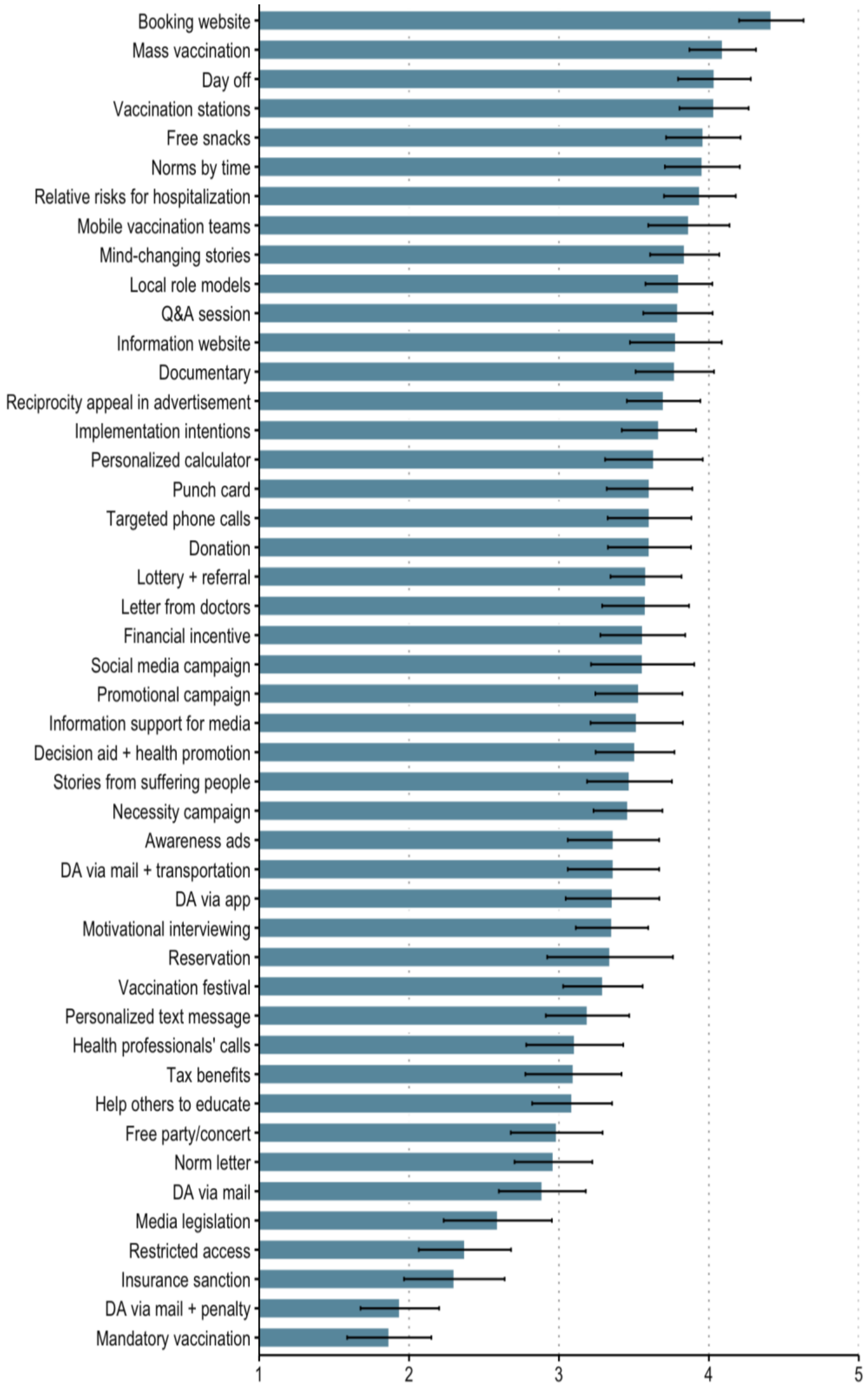

Figure S10. Expert ratings: Mean values in probability of non-pharmaceutical side-effects.

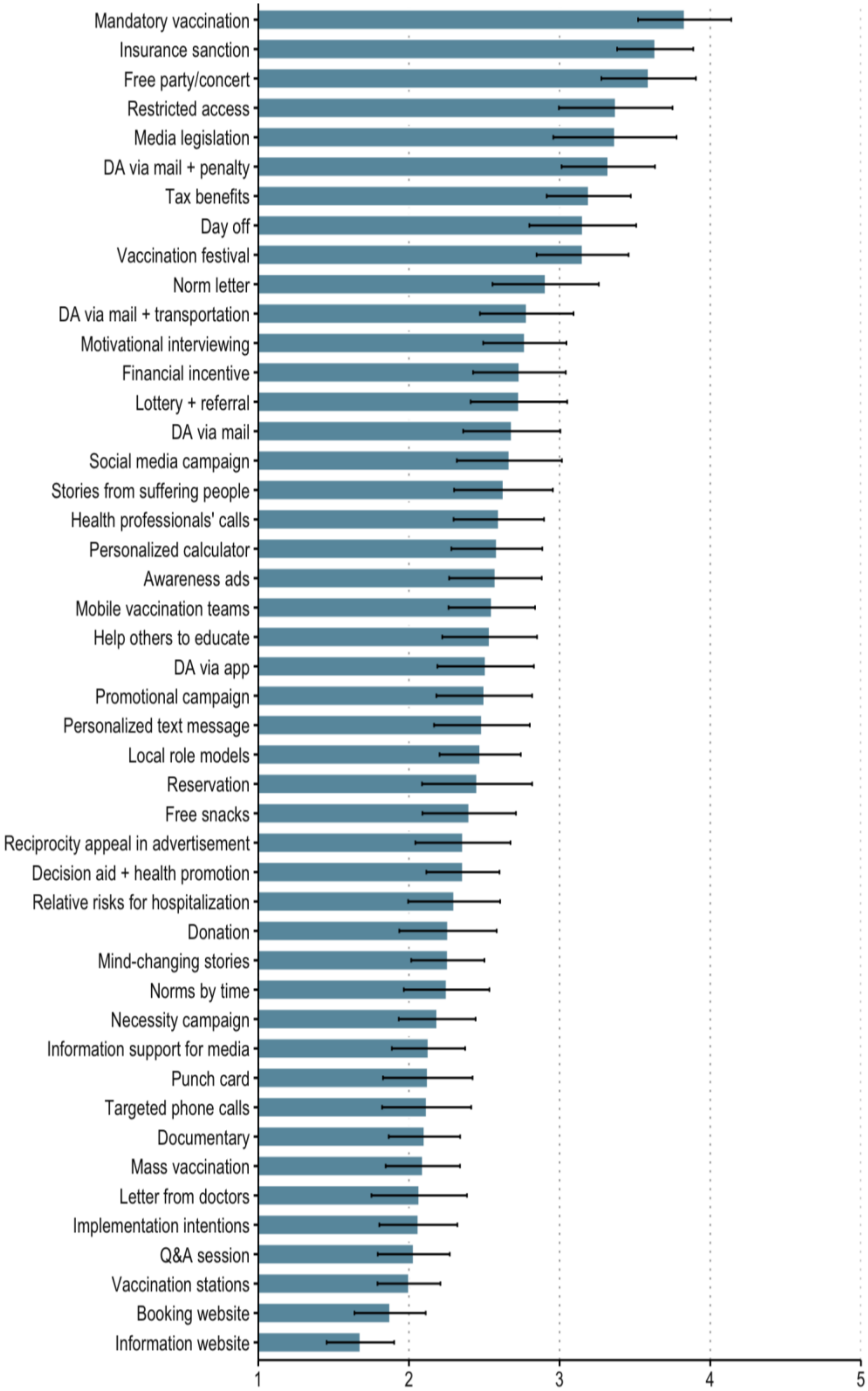

Figure S11. Expert ratings: Mean values in inequity.

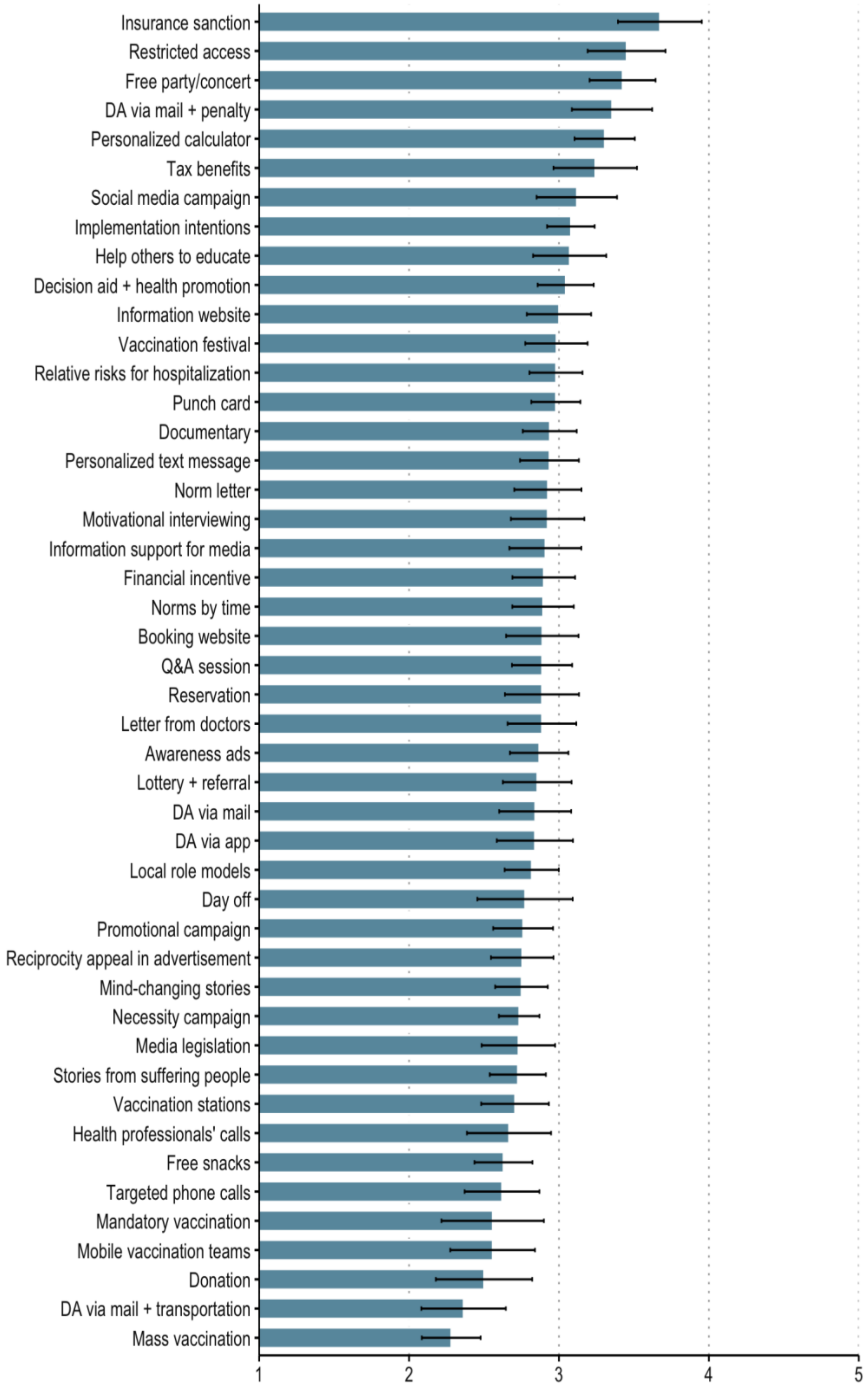

Figure S12. Expert ratings: Mean values in universality across countries.

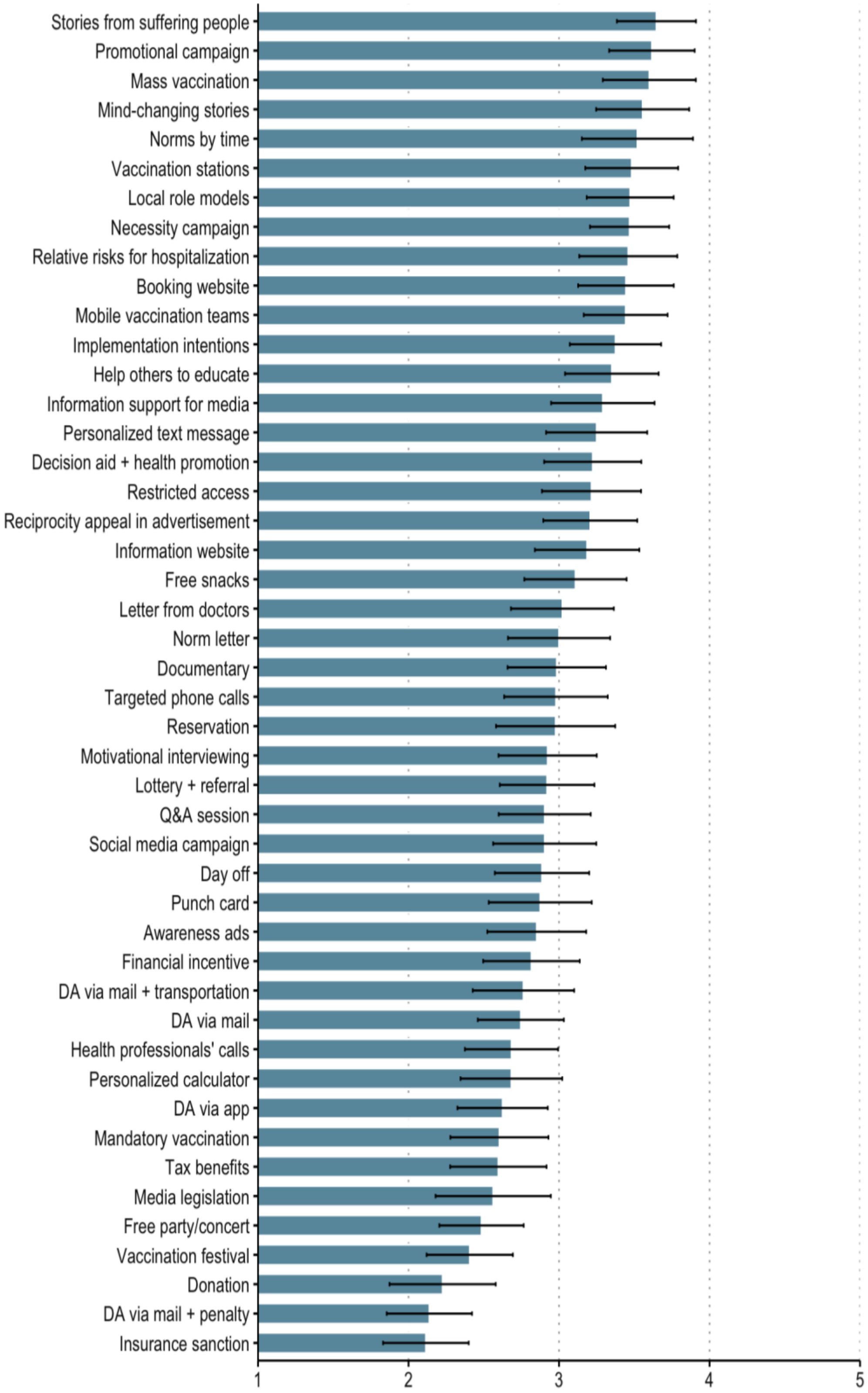

Figure S13. Expert ratings: Mean values in effect on previously unvaccinated people.

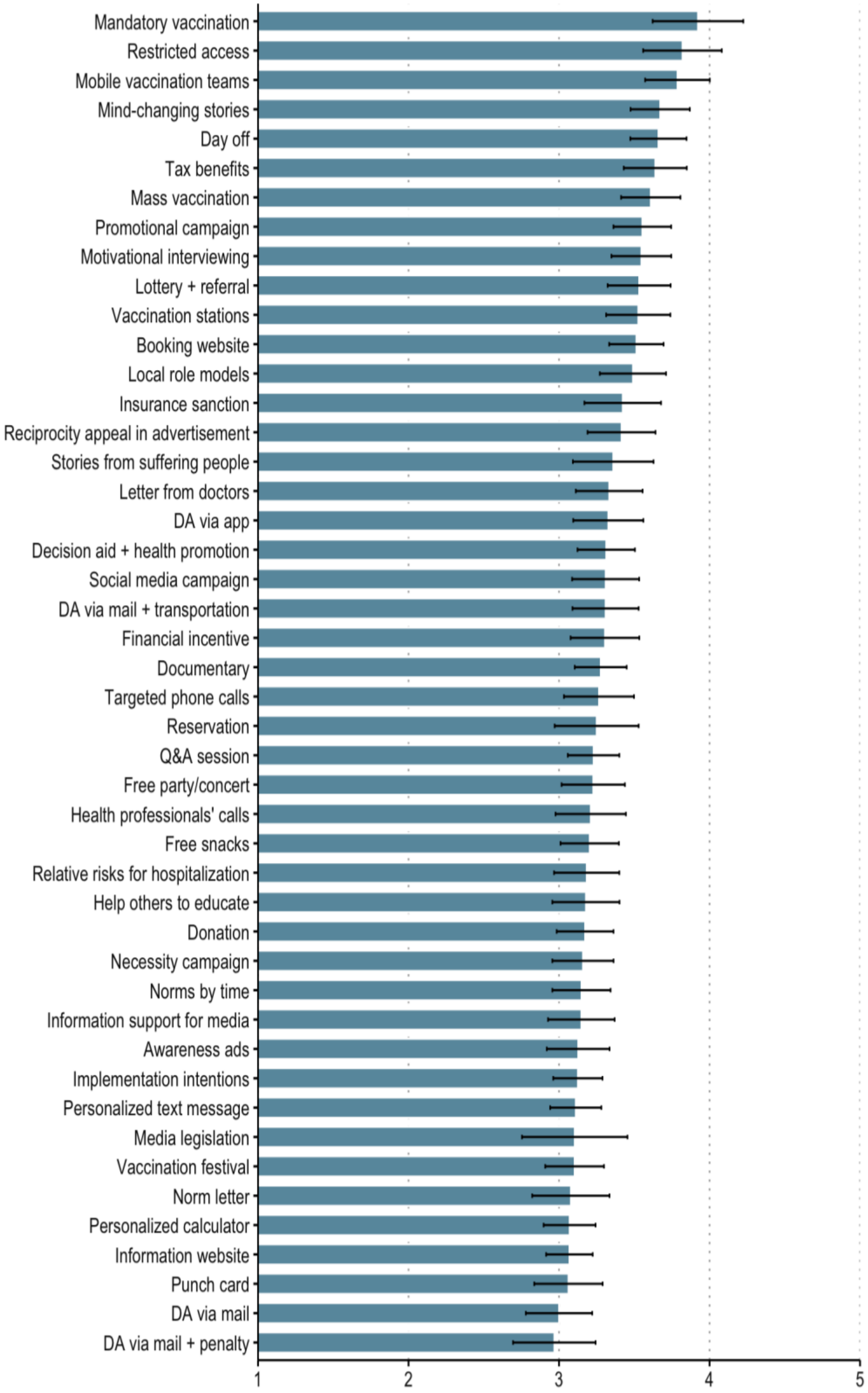

Figure S14. General population ratings: Mean values in effect on booster uptake

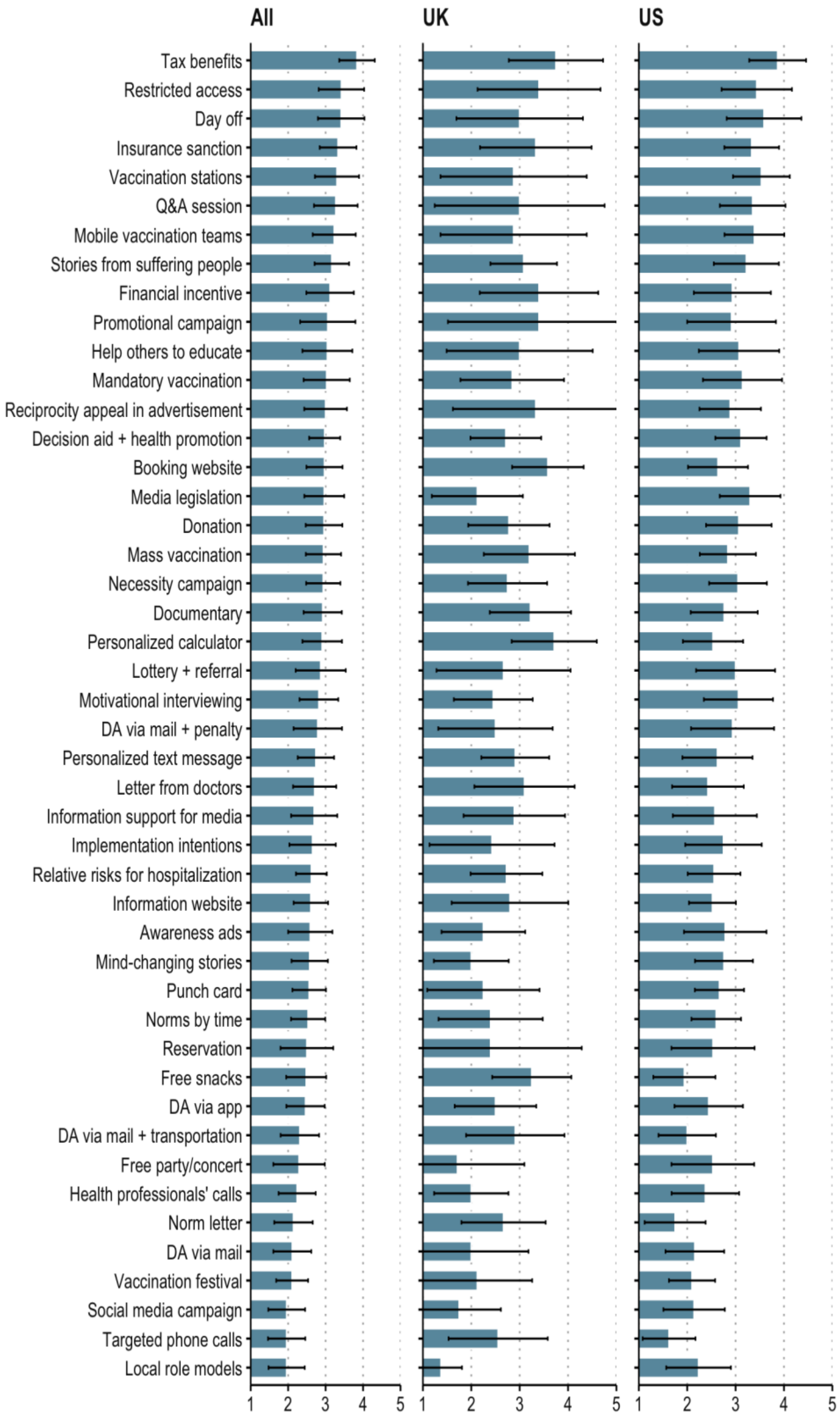

Figure S15. General population ratings: Mean values in effect on own booster intention. Based on subsample of participants who have not yet received a booster vaccine at the time of the study (n=144).

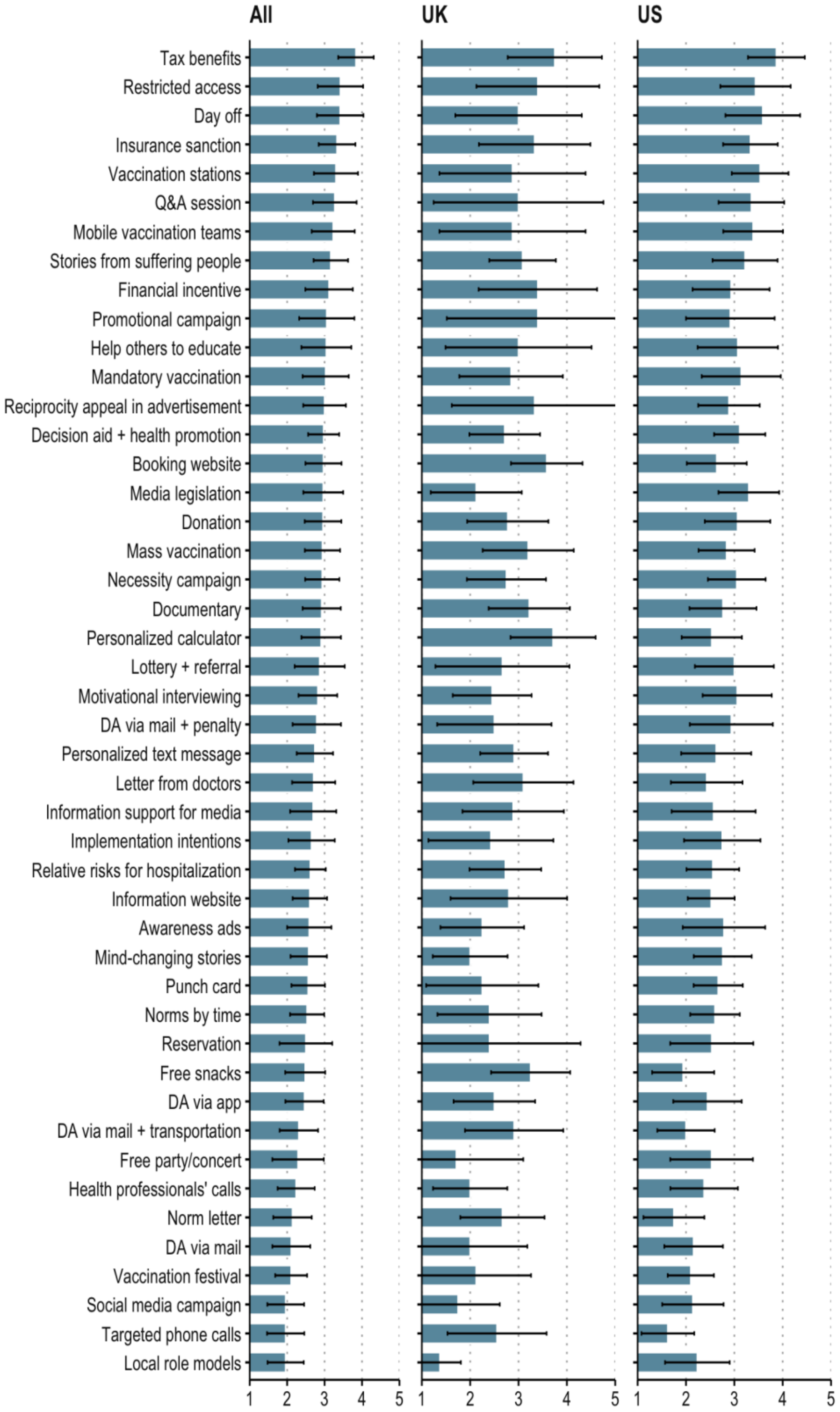

Figure S16. General population ratings: Mean values in perceived coercion.

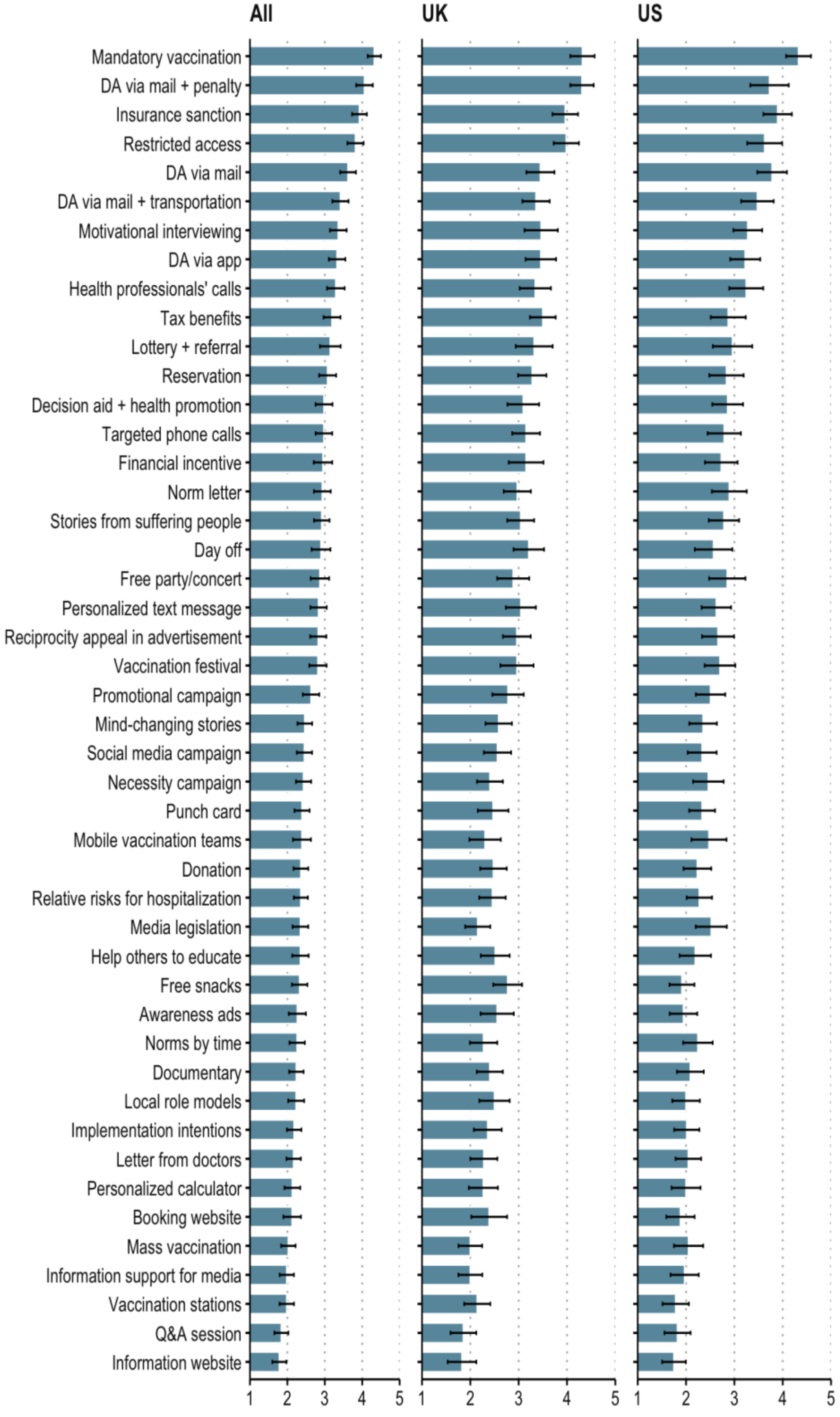

Figure S17. General population ratings: Mean values in reactance.

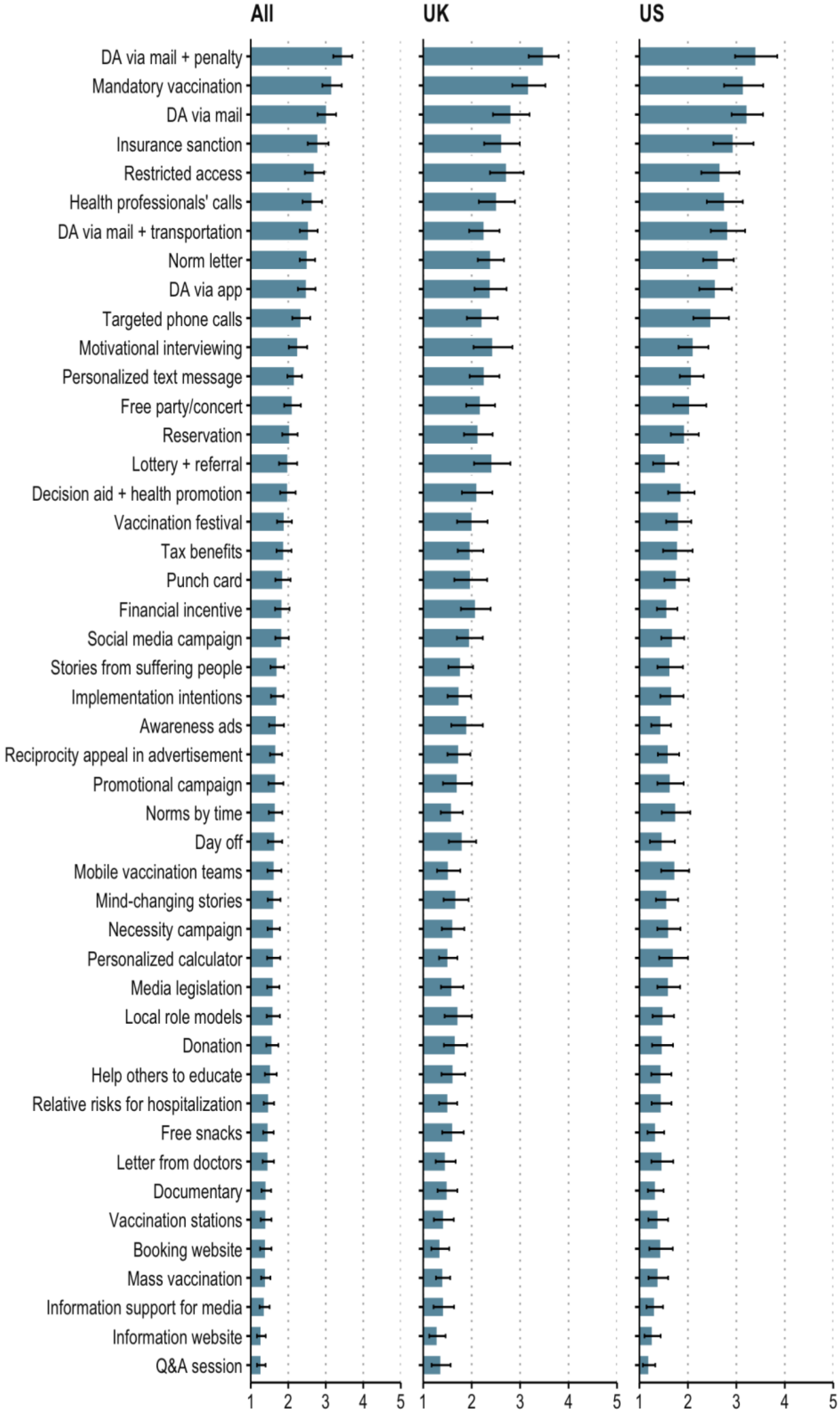

Figure S18. General population ratings: Mean values in acceptability for general population.

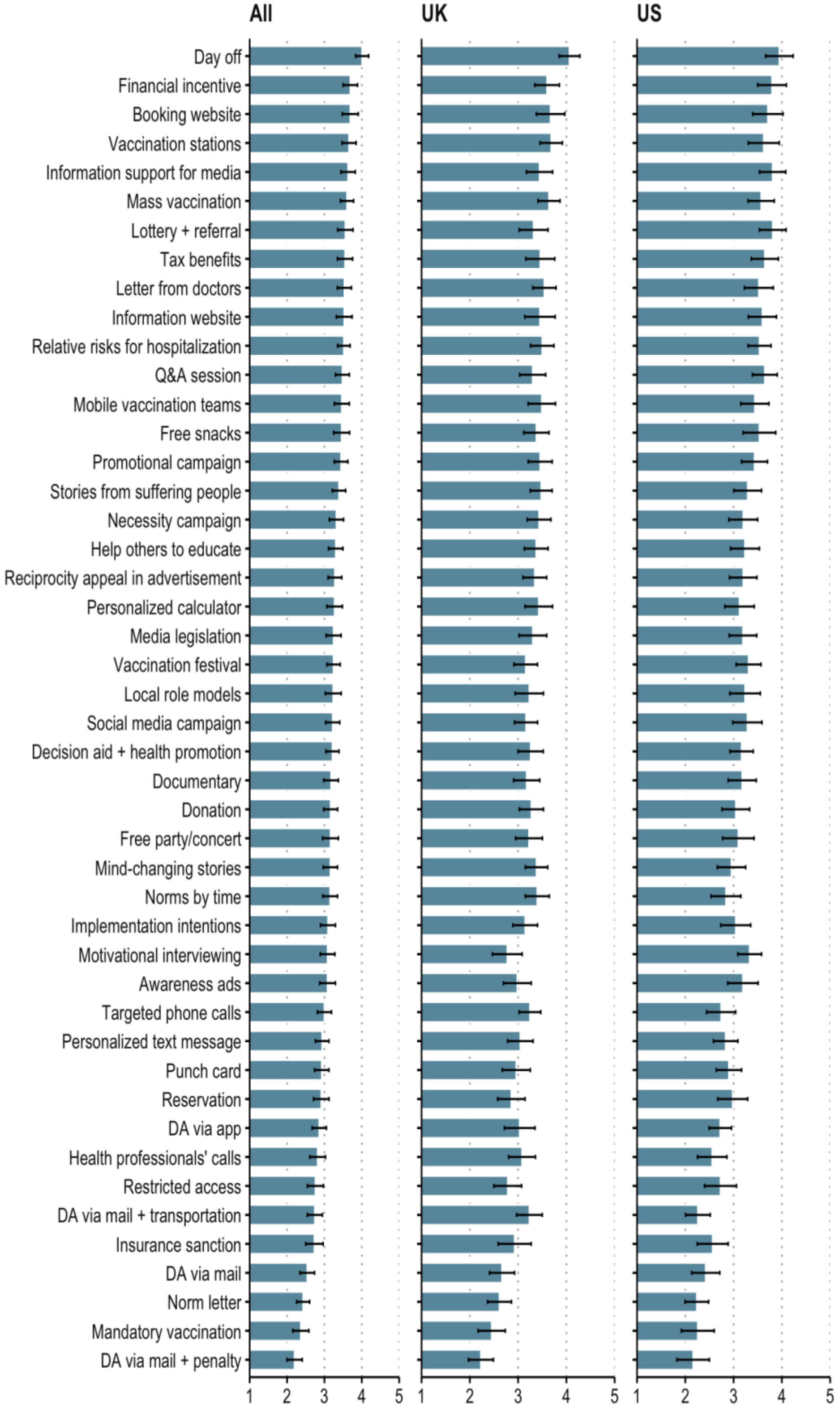

Figure S19. General population ratings: Mean values in activism intentions.

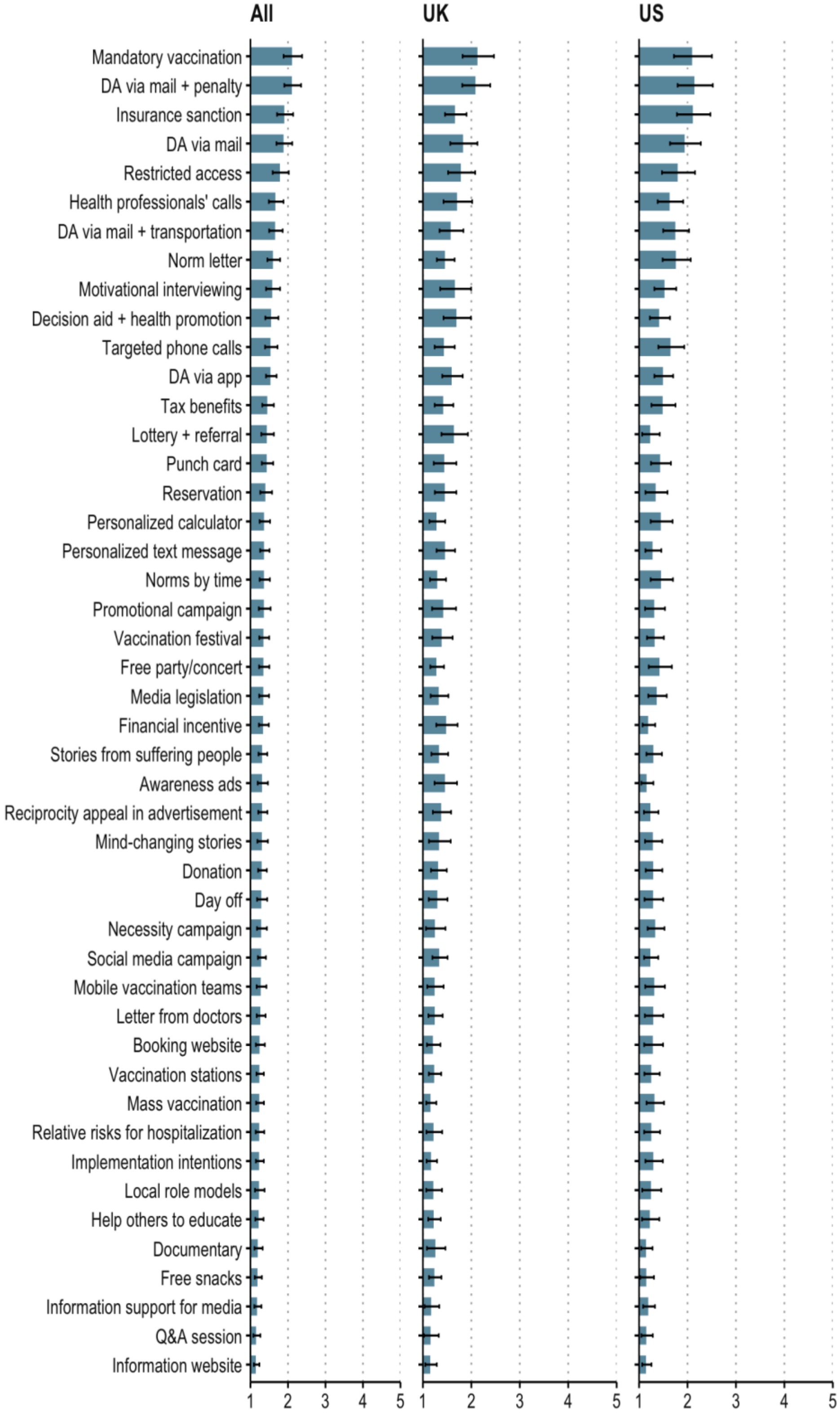

Supplement: Supplementary file 1 [file mmc1.pdf]
